# Diagnostic test accuracy of novel biomarkers for lupus nephritis—An overview of systematic reviews

**DOI:** 10.1371/journal.pone.0275016

**Published:** 2022-10-10

**Authors:** Juliana de Andrade Rebouças Guimarães, Silvania da Conceição Furtado, Ana Cyra dos Santos Lucas, Bruno Mori, José Fernando Marques Barcellos

**Affiliations:** 1 Coordenação de Medicina, Escola Superior de Ciências da Saúde, Universidade do Estado do Amazonas Manaus, Manaus, Amazonas, Brazil; 2 Departamento de Morfologia, Instituto de Ciências Biológicas, Universidade Federal do Amazonas, Manaus, Amazonas, Brazil; 3 Faculdade de Ciências Farmacêuticas, Universidade Federal do Amazonas, Manaus, Amazonas, Brazil; 4 Programa de Pós-graduação em Imunologia Básica e Aplicada, Instituto de Ciências Biológicas, Universidade Federal do Amazonas, Manaus, Amazonas, Brazil; University of Utah School of Medicine, UNITED STATES

## Abstract

**Introduction:**

Systemic lupus erythematosus (SLE) is a chronic autoimmune disease with multiorgan inflammatory involvement and a mortality rate that is 2.6-fold higher than individuals of the same age and sex in the general population. Approximately 50% of patients with SLE develop renal impairment (lupus nephritis). Delayed diagnosis of lupus nephritis is associated with a higher risk of progression to end-stage renal disease, the need for replacement therapy, and mortality. The initial clinical manifestations of lupus nephritis are often discrete or absent and are usually detected through complementary tests. Although widely used in clinical practice, their accuracy is limited. A great scientific effort has been exerted towards searching for new, more sensitive, and specific biomarkers in recent years. Some systematic reviews have individually evaluated new serum and urinary biomarkers tested in patients with lupus nephritis. This overview aimed to summarize systematic reviews on the accuracy of novel serum and urinary biomarkers for diagnosing lupus nephritis in patients with SLE, discussing how our results can guide the clinical management of the disease and the direction of research in this area.

**Methods:**

The research question is “*What is the accuracy of the new serum and urinary biomarkers studied for the diagnosis of LN in patients with SLE*?*”*. We searched for systematic reviews of observational studies evaluating the diagnostic accuracy of new serum or urinary biomarkers of lupus nephritis. The following databases were included: PubMed, EMBASE, BIREME/LILACS, Scopus, Web of Science, and Cochrane, including gray literature found via Google Scholar and PROQUEST. Two authors assessed the reviews for inclusion, data extraction, and assessment of the risk of bias (ROBIS tool).

**Results:**

Ten SRs on the diagnostic accuracy of new serum and urinary BMs in LN were selected. The SRs evaluated 7 distinct BMs: (a) antibodies (anti-Sm, anti-RNP, and anti-C1q), (b) cytokines (TWEAK and MCP-1), (c) a chemokine (IP-10), and (d) an acute phase glycoprotein (NGAL), in a total of 20 review arms (9 that analyzed serum BMs, and 12 that analyzed BMs in urine). The population evaluated in the primary studies was predominantly adults. Two SRs included strictly adults, 5 reviews also included studies in the paediatric population, and 4 did not report the age groups. The results of the evaluation with the ROBIS tool showed that most of the reviews had a low overall risk of bias.

**Conclusions:**

There are 10 SRs of evidence relating to the diagnostic accuracy of serum and urinary biomarkers for lupus nephritis. Among the BMs evaluated, anti-C1q, urinary MCP-1, TWEAK, and NGAL stood out, highlighting the need for additional research, especially on LN diagnostic panels, and attempting to address methodological issues within diagnostic accuracy research. This would allow for a better understanding of their usefulness and possibly validate their clinical use in the future.

**Registration:**

This project is registered on the International Prospective Registry of Systematic Reviews (PROSPERO) database (CRD42020196693).

## Introduction

Systemic lupus erythematosus (SLE) is a chronic autoimmune disease with multiorgan inflammatory involvement. The mortality rate for individuals with SLE is 2.6-fold higher than that the same age and sex in the general population [1]. Approximately 50% of patients with SLE develop renal impairment, i.e., lupus nephritis (LN) [2–4]. LN consists of renal alterations that can compromise the glomerulus, interstitium, tubules, and blood vessels, with different severities and combinations [2]. The great importance of LN lies in the significant number of affected patients and the potential to directly influence patient prognosis [5, 6].

The mortality is higher in patients with LN than in those without lupus renal impairment, being as high as 25% among those with severe proliferative forms of the disease (class III and IV) [7, 8].

Treatment for LN has drastically changed patient survival in recent years. However, 10 to 30% of patients still progress to end-stage renal disease and require dialysis and transplantation [9, 10].

The gold standard for diagnosing LN is renal biopsy. Kidney histopathology allows (a) the stratification of LN based on the World Health Organization (WHO) classification modified by the Renal Pathology Society/International Society of Nephrology Working Group on the Classification of Lupus Nephritis (RSP/ISN 2003) [10–12]; (b) the evaluation of the presence of active and chronic inflammatory lesions (activity and chronicity indices of the National Institutes of Health—NIH) [13]; (c) the verification of the presence of disease in other renal compartments—such as the vascular and tubulointerstitial compartments; (d) and the identification of other coexistent lesions, whether autoimmune or not (e.g., IgA nephropathy, diabetic nephropathy, hypertensive nephropathy, etc.).

However, the biopsy is not widely available in all health services; it requires infrastructure, training and carries the risk of complications, such as hematuria, damage and loss of the biopsied kidney, or even death [14]. Therefore, biopsies are performed for diagnosis, not for frequent routine monitoring, with repeat biopsy being reserved for patients with unexpected clinical course, relapse, distinction between LN activity and cronicity, and to rule other concomitant diseases.

Accordingly, monitoring LN is achieved using the following biomarkers adopted by the international guidelines for lupus nephritis management: anti-DNAds, serum complement levels (C3 and C4), creatinine clearance, urinalysis with urine sediment microscopy, and proteinuria, represented by 24-hour proteinuria or protein/creatinine ratio in an isolated urine sample [15, 16].

Unfortunately, their accuracy is limited [7, 17], compromising the distinction between active and chronic renal lesions and the differentiation between LN and comorbidities that may be concomitant in an individual SLE patient. In addition, studies that have evaluated the possible advantages of performing programmed repetition of kidney biopsies showed clinicopathological dissociation. Malvar et al. observed that one-third of patients who had achieved clinical remission of nephritis had active inflammatory lesions in the histopathological analysis of the kidney and that 62% of individuals considered to have active kidney disease were in histopathological remission [18].

Delayed diagnosis of LN is associated with a higher risk of progression to end-stage renal disease, the need for replacement therapy, and mortality [19]. Thus, improving the prognosis of these patients involves early detection of the disease, definition of its severity, and prediction of its response to treatment and relapse.

In recent years, a great scientific effort has been exerted in the search for new biomarkers. Several studies suggest possible candidates, such as genes [20–23], antibodies [24–26], cytokines [27, 28], chemokines [27], adhesion molecules [29–31], growth factors [32, 33], cell surface molecules [34], and cell populations [35, 36], among others.

Some systematic reviews (SRs) have individually evaluated new serum and urinary biomarkers tested in patients with LN, for example, neutrophil gelatinase-associated lipocalin (NGAL) [37], monocyte chemoattractant protein 1 (MCP-1) [38], and interferon-inducible protein 10 (IP-10) [39]. In a preliminary search in the PubMed database, although several narrative reviews address this subject, no overviews were found.

This study aimed to summarize SRs on the accuracy of novel serum and urinary biomarkers for diagnosing LN in patients with SLE. The research question is "*What is the accuracy of the novel serum and urinary biomarkers studied for the diagnosis of LN in patients with SLE*?*"*.

## Methods

### Protocol and registration

The protocol for this overview was registered in August 2020 on the Prospero platform of the University of York under the number **CRD42020196693**.

### Selection criteria

#### Type of studies

Systematic Reviews (SR), with or without meta-analyses, of observational studies evaluating the diagnostic accuracy of serum or urinary new biomarkers of LN were included.

#### Participants

Participants in the included studies were patients diagnosed with SLE, classified by the ACR (1997), SLICC (2012), or ACR/EULAR 2019 criteria, in the outpatient or in-hospital settings, without sex or age restrictions.

#### Index test

Studies evaluating new serum and urinary biomarkers, or combinations of these biomarkers (biomarker panels) tested for the detection of LN (diagnosis, activity monitoring, prediction of flare, and severity) were included.

#### Reference test

Currently, the reference tests used in clinical practice include anti-DNAds, C3, C4, creatinine clearance, urinalysis with sediment microscopy, 24-h proteinuria or protein/creatinine ratio in an isolated urine sample, and renal biopsy. These biomarkers are considered standard by the *European Alliance of Associations for Rheumatology* (EULAR) and the *American College of Rheumatology* (ACR). They are widely used for the detection and monitoring of LN.

Primary studies evaluating the diagnostic accuracy of LN biomarkers usually use a combination of tests to define the presence of nephritis. Given this peculiarity of this field of research, this overview considered all SRs of studies that evaluated new biomarkers by comparing patients with and without LN, patients with active and inactive LN, patients with renal relapse, and without renal relapse, and patients with proliferative and non-proliferative LN using any combination of those tests as reference.

#### Outcome measures

The primary outcome was the diagnostic accuracy of each biomarker to identify LN in patients with SLE. The secondary outcomes of interest were the diagnostic accuracy for detecting active LN, prediction of renal relapse, identification of response to treatment, and differentiation between proliferative and nonproliferative LN forms.

#### Exclusion criteria

SRs evaluating biomarkers for detecting only other clinical manifestations of disease activity in SLE; those that did not describe the quantitative data relative to diagnostic accuracy of the test assessing the biomarkers; and those evaluating only genetic biomarkers (search for genes and variants), imaging and histopathological techniques were excluded. Primary studies, case reports, narrative reviews, and other types of publications, such as editorials, comments, and letters were excluded as well.

### Literature search

The databases used to search for evidence were PubMed, EMBASE, BIREME/LILACS, Scopus, Web of Science, and Cochrane, including gray literature found through Google Scholar and PROQUEST, from inception until April 2022. The search strategy was developed based on the PIRD (Population, Index test, Reference test, Diagnosis) approach with an information specialist, using free-text and subject headings referring to “SLE” OR “LN”, AND “biomarkers”. The type of study was not included in the search strategy to increase its sensitivity. **S1 Table** provides the search strategy constructed for all databases searched. This strategy was adapted to the other databases. No language restriction was applied.

### Selection of studies

The selection of studies was performed by two reviewers (JARG and BM) after the removal of duplicates using EndNoteX9. This process was done in two stages. In the first stage, studies were selected based on titles and abstracts, and in the second stage, studies were selected based on full text analysis, checking the eligibility criteria. Disagreements were resolved by consensus and, in case of persistent discrepancies, the decision was made by a third reviewer (JFMB).

### Data extraction and management

Data were extracted by two authors (JARG and BM), into a table containing the following information: review question, objectives, population (characteristics, total number), clinical context (outpatient, hospital), index biomarker, reference biomarker, biological material, technique used, details of the search, outcome, databases searched, date range of included studies, number of included studies, methodological quality assessment tool, diagnostic accuracy results, heterogeneity, publication bias, and conclusion.

### Data analysis

Extracted data were analyzed by three reviewers (JARG, JFMB, and SCF), qualitatively summarized, and presented in tables. Data from selected SRs were reported as diagnostic accuracy measures: pooled sensitivity, pooled specificity, positive likelihood ratio (PLR), negative likelihood ratio (NLR), diagnostic odds ratio (DOR), and summary ROC curve area under the curve (SROC-AUC).

It was reported when more than one SR evaluating the same biomarker presented similar conclusions. When conflicting results existed, the possible reasons were explored.

#### Assessment of reporting bias

The Deeks test was used to investigate possible publication bias, if possible. Despite the limitations of the evaluation of this aspect in systematic reviews of diagnostic tests accuracy, the likelihood of publication bias was reduced by the extensive search of studies in the databases already cited, in the grey literature, hand searching the references, and by including conference proceedings.

### Assessment of methodological quality

The risk of bias of the included reviews was analyzed by two reviewers (JARG e BM) using the ROBIS tool [40]. Any disagreements were judge by a third author (ACSL).

## Results

**Fig 1:**
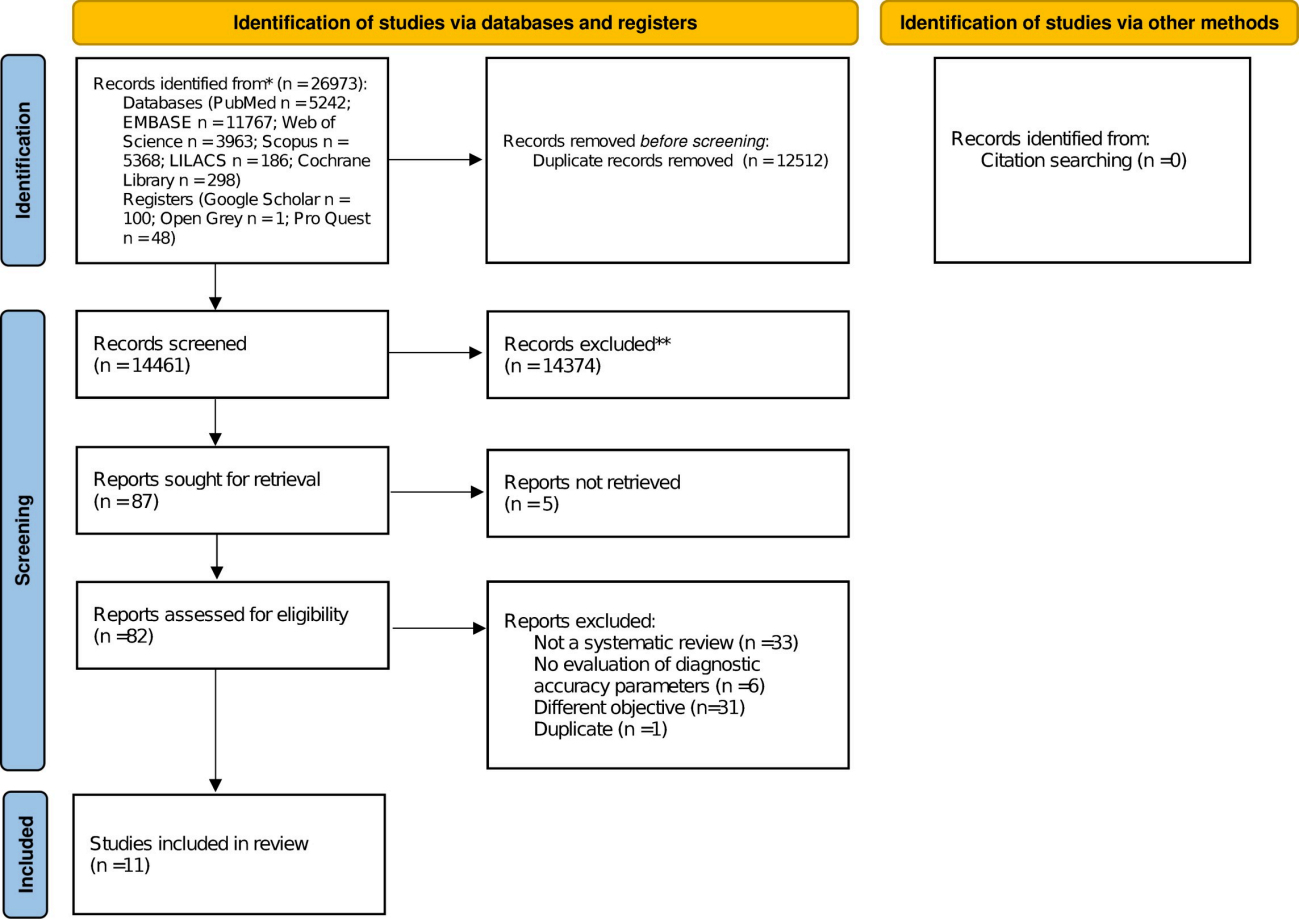
Overview flow-diagram.

In total, 26,973 articles addressing biomarkers (BMs) in lupus nephritis (LN) were identified. After exportation to EndNote, 12,512 duplicates were detected and removed. During Phase 1, 14,461 articles were evaluated by titles and abstracts, leaving 87 articles for full-text analysis. Finally, 10 systematic reviews (SRs) met the eligibility criteria and were included in this overview, as shown in **Fig 1**.

### Description of the included reviews

Ten SRs on the diagnostic accuracy of new serum and urinary BMs in LN were selected. The SRs evaluated 7 distinct BMs in a total of 20 review arms (9 that analysed serum BMs [39, 41–46], and 12 that analysed BMs in urine [37, 39, 43, 44, 47–49], as shown in **Table 1**. The population evaluated in the primary studies was predominantly adults. Two SRs included strictly adults, 5 reviews also included studies in the paediatric population, and 4 did not report the age groups (**Table 2**).

**Table 1:** Overview of key characteristics of included reviews.

Author (year)	Country	Search date	Population	Index test	Biological sample	Tipo de BM	Reference test	Diagnosis	Meta-analysis	Number of included studies	Study design
Benito-Garcia, E et al. (2004)	USA	January 1966—December 2003	SLE patients	anti-Sm	serum	antibody	Renal biopsy; clinical parameters	LN	No	13 (8 metanalysed)	NI
			SLE patients	anti-RNP	serum	antibody	Renal biopsy; clinical parameters	LN	No	8	NI
Yin, Y. et al. (2012)	China	Until October 2011	SLE patients	anti-C1q	serum	antibody	Renal biopsy; clinical parameters	LN	Yes	7	NI
			LN patients	anti-C1q	serum	antibody	Renal biopsy; clinical parameters	LN activity	Yes	22	NI
Eggleton P. et al. (2014)	United Kingdom	1977–2013	SLE patients	anti-C1q (ELISA)	serum	antibody	Renal biopsy; clinical parameters	LN	Yes	25 (22 meta-analysed)	NI
			LN patients	anti-C1q (ELISA)	serum	antibody	Renal biopsy; clinical parameters	LN activity	Yes	31 total (28 meta-analysed)	NI
Fang Y. G. et al. (2015)	China	Until December 2014	SLE patients	uNGAL	urine	acute phase glycoprotein	Renal biopsy	LN	Yes	4	CS
			LN patients	uNGAL	urine	acute phase glycoprotein	SLEDAI; SLICC; BILAG-2004; SLEDAI 2000;	LN activity	Yes	8	3 CS; 5 PC
			LN patients	uNGAL	urine	acute phase glycoprotein	SLEDAI; SLEDAI 2000; BILAG-2004; clinical parameters	Prediction of LN flares	Yes	6	1 CS; 5 PC
Wang, Z. et al. (2015)	China	Until March 2015	SLE patients	anti-C1q	serum	antibody	Renal biopsy; clinical parameters	LN	Yes	3	NI
Puapatanakul, P; et al. (2019)	Thailand	Until December 2017	SLE patients	IP-10	serum	chemokine	SLEDAI; BILAG; SLAM-R renal biopsy; SELENA-SLEDAI; clinical parameters	LN	No	2	NI
			SLE patients	IP-10	urine	chemokine	SLEDAI; BILAG; SLAM-R renal biopsy; SELENA-SLEDAI; clinical parameters	LN	No	5	NI
Gao, Y; et al. (2020)	China	Until October 2019	SLE patients	uNGAL	urine	acute phase glycoprotein	Renal biopsy	LN	Yes	6	PC
			LN patients	uNGAL	urine	acute phase glycoprotein	R-SLEDAI; BILAG2004; SLICC; BAI; clinical parameters	LN activity	Yes	9	7 CS; 2 PC
			LN patients	uNGAL	urine	acute phase glycoprotein	R-SLEDAI; BILAG2004; pBILAG; clinical parameters	LN prediction of flare	Yes	10	3 CS; 7 PC
			LN patients	uNGAL	urine	acute phase glycoprotein	Renal biopsy	Proliferative LN	Yes	6	PC
Wang, Z. et al. (2020)	China	Until September 2019	SLE patients	TWEAK	urine	cytokine	Renal Biopsy; clinical paranmeters; R-SLEDAI	LN	Yes	11	NI
			SLE patients	TWEAK	urine	cytokine	Renal Biopsy; clinical paranmeters; R-SLEDAI	LN activity	Yes	4	NI
Xia, Y-R. et al. (2020)	China	Until November 2019	LN patients	MCP-1	urine	cytokine	SLEDAI	LN activity	Yes	3	NI
Ma, H. Y. et al. (2021)	China	Until August 2020	SLE patients	TWEAK	urine and serum	cytokine	Renal Biopsy; R-SLEDAI	LN activity	Yes	9	8 CS; 1 PC

NI = Not informed; *Clinical parameters = 24h proteinuria, Urine Protein to Creatinine Ratio (UPCR), creatinine, active sediment; ^a^ R-SLEDAI = Renal-Systemic lupus erythematosus disease activity index; ^b^ SLEDAI-2000 = Systemic lupus erythematosus disease activity index 2000; ^c^ SLEDAI = Systemic lupus erythematosus disease activity index; ^d^ SLICC = The Systemic Lupus International Collaborating Clinics; ^e^ BAI = Biopsy activity index; ^f^ BILAG 2004 = British Isles Lupus Assessment Group’s disease activity index; ^g^ pBILAG = Pediatric British Isles Lupus Assessment Group index; ^h^SLICC/ACR DI = Systemic Lupus International Collaborating CLinics/American College of Rheumatology Criteria Damage Index; ^i^R-BILAG = Renal British Isles Lupus Assessment Group; ^j^SLICC RAS = The Systemic Lupus International Collaborating Clinics Renal Activity Score; ^l^SLAM = Systemic lupus activity measure; ^m^ CS = Cross-sectional; ^n^PC = prospective cohort; ^o^BM = biomarkers; ^p^CC = case-control; ^q^LS = longitudinal study.

**Table 2:** Summary of principal data of included reviews.

Author (year)	Index test	Number of included studies (number of participants)	Subjects age (years)	Diagnosis	Pooled sensitivity	Pooled specificity	PLR	NLR	SROC-AUC	DOR	Heterogeneity	Publication bias
Benito-Garcia, E et al. (2004)	anti-Sm	8 (n = 984)	NR	LN	0.25 (0.17–0.36)	0.85 (0,78–0.91)	1.3	NR	NR	NR	NR	NR
	anti-RNP	8 (n = 1114)	NR	LN	0.28 (0.18–0.41)	0.74 (0.65–0.81)	1.1	NR	NR	NR	NR	NR
Yin, Y. et al. (2012)	anti-C1q	22 (n = 2381)	9,8–43	LN	0,58 (0,56–0,61)	0,75 (0,72–0,77)	2,6 (2,06–3,28)	0,51 (0,41–0,63)	0,7941	6,08 (3,91–9,47)	High	Yes
	anti-C1q	9 (n = 517)	9,8–43	activity	0,74 (0,68–0,79)	0,77 (0,71–0,82)	2,91 (1,83–4,65)	0,33 (0,19–0,56)	0,8378	10,56 (4,56–24,46)	High	Yes
Eggleton P. et al. (2014)	anti-C1q	31 (28 meta-analysed) (n = 2769)	> = 15 anos (15–77); pediátrico (mean age 13,9)	LN	0.73	0.70 (0.57–0.81)*	2.66	0.40	NR	NR	NR	NR
	anti-C1q	31 (9 meta-analysed) (n = 517)	> = 15 anos (15–77); pediátrico (mean age 13,9)	activity	0.80	0.75 (0.46–0.91)*	3.79	0.30	NR	NR	NR	NR
Fang Y. G. et al. (2020)	uNGAL	4 (n = 177)	10–35	LN	0.73 (0.61–0.83)	0.78 (0.69–0.85)	3.88 (1.14–13.24)	0.36 (0.160–0.82)	0.86	14.83	Moderate to High	No
	uNGAL	8 (n = 815)	11,6–44,1	activity	0.66 (0.60–0.71)	0.62 (0.57–0.66)	2.05 (1.25–3.37)	0.43 (0.22–0.86)	0.75	5.46	HIgh	No
	uNGAL	6 (n = 442)	14,1–41	Prediction of flares	0.77 (0.68–0.85)	0.65 (0.60–0.70)	2.24 (1.47–3.42)	0.37 (0.17–0.81)	0.77	6.28	Moderate	No
Wang, Z. et al. (2015)	anti-C1q	11 (n = 1084)	9–37,1 (+/- 11,9)	LN	0,67 (0,63–0,71)	0,69 (0,65–0,74)	2,18 (1,75–2,72)	0,48 (0,39–0,60)	0,749	5,09 (3,29–7,85)	Moderate	Yes
Puapatanakul, P; et al. (2019)	Serum IP-10	2 (n =?)	NR	LN	QS	QS	QS	QS	QS	QS	QS	QS
	Urinary IP-10	5 (n =?)	NR	LN	QS	QS	QS	QS	QS	QS	QS	QS
Gao, Y; et al. (2020)	uNGAL	9 (n = 573)	11,6–35	LN	0,84 (95% CI 0,71–0,91)	0,91 (95% CI 0,70–0,98)	9,08 (95% CI 2,31–35,69)	0,18 (95% CI 0,09–0,35)	0,92 (95% CI 0,90–0,94)	50,51 (95% CI 8,15–313,03)	High	No
	uNGAL	10 (n = 949)	11,6–44,1	activity	0,72 (0,56–0,84)	0,71 (0,51–0,84)	2,45 (1,32–4,54)	0,39 (0,22–0,70)	0,77 (0,74–0,81)	6,24 (2,08–18,68)	High	No
	uNGAL	6 (n = 442)	11,3–41	prediction of flare	0,80 (0,57–0,92)	0,67 (0,58–0,75)	2,41 (1,57–3,72)	0,30 (0,11–0,79)	0,74 (0,70–0,78)	8,08 (2,02–32,35)	Moderate	No
	uNGAL	2 (n = 36)	10–30	Proliferative LN	0,87 (0,66–0,97)	0,69 (0,39–0,91)	2,89 (1,26–6,61)	0,20 (0,06–0,65)	not constructed	16,42 (2,56–105,37)	NR	NR
Wang, Z. et al. (2020)	TWEAK	4 (n = 276)	28 (+/- 11,8) - 35,5 (+/- 12,7)	LN	0,55 (0,47–0,63)	0,92 (0,86–0,96)	NR	NR	0,8224	16,54 (7,57–36,15)	Low	No
	TWEAK	3 (n = 139)	25,6 (+/- 10,7) - 32,9 (+/- 10,37)	activity	0,91 (0,82–0,96)	0,70 (0,58–0,81)	NR	NR	0,8131	18,54 (7,45–45,87)	Low	No
Xia, Y-R. et al. (2020)	MCP-1	7 (n = 521)	23,66 (+/-4,55) - 36,9 (+/-10,62)	activity	0,89 (0,86–0,93)	0,63 (0,55–0,69)	2,16 (1,66–2,80)	0,15 (0,08–0,30)	0,90	19,40 (7,24–51,96)	Moderate to High	No
Ma, H. Y. et al. (2021)	TWEAK	9 (n = 334)	NR	activity	0,69 (0,63–0,75)	0,77 (0,71–0,82)	3,31 (2,05–5,35)	0,38 (0,26–0,55)	0,827	10,89 (6,73–17,63)	High	No

* median; QS = Qualitative synthesis; NR = Not reported; BM = biomarkers

The SRs included observational cross-sectional studies (23 studies), and cohort studies (22 studies). Seven SRs (12 arms) did not report the designs of the included primary studies. The main outcomes evaluated were the accuracy of the BM for the diagnosis of LN in patients with systemic lupus erythematosus (SLE) [37, 39, 41–43, 45–48], the detection of disease activity [37, 42, 43, 46–49], the prediction of renal relapse [37, 47] and the stratification of severity [37]. No review evaluated prognosis or early response to treatment. Only 4 SRs [37, 42, 43, 47] informed the cutoff thresholds for positivity among primary studies. We summarized those in the **S2 Table**. The main characteristics and data of the included SRs are shown in **Tables 1** and **2.**

### Biomarkers studied

*Autoantibodies*. Several autoantibodies have been investigated as possible BMs in LN. We found 4 SRs that evaluated the diagnostic accuracy of the following autoantibodies in LN: anti-Sm, anti-RNP [41] and anti-C1q [42, 45, 46].

*Anti-Sm and anti-RNP*. Anti-Sm and anti-RNP are autoantibodies that target small nuclear ribonucleoproteins (snRNPs); they are among the most used BMs in patients with diagnosed or suspected systemic autoimmune diseases [50].

Anti-Sm is associated with the diagnosis of SLE and is part of the disease classification criteria [51]. However, the role of anti-Sm has been investigated in other contexts and has been associated with other clinical variables of the disease, such as pericarditis, CNS involvement and renal involvement [52–55].

Anti-RNP antibodies can be detected in several systemic autoimmune diseases, including SLE. However, its clinical value is found in the strong association of high titres with mixed connective tissue disease (MCTD) [50].

Benito-Garcia, et al. conducted a systematic review to determine the sensitivity, specificity and predictive values of anti-Sm and anti-RNP autoantibodies in the diagnosis of SLE and other related systemic autoimmune diseases, and to identify their clinical associations.

Thirteen studies were included in this SR, which evaluated the accuracy of anti-Sm antibodies in the detection of LN. Additionally, 8 of these studies were included in a meta-analysis, totalizing 984 patients. The weighted mean sensitivity was 0.25 (95% CI 0.17–0.36), the specificity was 0.85 (95% CI 0.78–0.91), and the median PLR was 1.3. The corresponding summary receiver operating characteristics (SROC) showed that most of the points were dispersed around the diagonal line, which, together with the reported data, demonstrate the low relevance of this BM as a potential influencer of clinical decision-making in this context.

The 5 studies that were not included in the meta-analysis were qualitatively synthesized [56–60]. In 3 of these studies [56, 58, 59], no significant correlation was found between anti-Sm and renal involvement of the disease. One of the studies correlated anti-Sm with WHO Class V nephritis (membranous glomerulonephritis) [57]. The other study by Win et al., only 1 of the 23 lupus patients who were positive for anti-Sm presented Class IV nephritis (diffuse proliferative glomerulonephritis), and among the other patients, most presented mesangial, membranous or focal histopathological changes, and 4 had a normal renal biopsy [60].

Eight of the included studies analysed the value of anti-RNP antibodies in the diagnosis of LN. The meta-analysis showed the following weighted mean results: sensitivity was 0.28 (95% CI 0.18–0.41), specificity was 0.74 (95% CI 0.65–0.81), and PLR was 1.1. The SROC also showed the dispersion of the points around the diagonal line, reinforcing the conclusion that this antibody is of little use in the detection of LN.

In both arms of this SR (anti-Sm and anti-RNP), the quality of the studies was evaluated using the criteria developed by the Evidence-based Medicine Working Group [61], and only studies classified as Grade A and Grade B (high methodological quality) were included in the reviews. However, the presence of heterogeneity among the studies in either of the 2 arms of the SRs was not evaluated, and it was not possible to analyse the impact of such heterogeneity on the results. Furthermore, only research reports in English were included.

*Anti-C1q*. Although it was initially described in the serum of SLE patients, anti-C1q autoantibodies have been detected in up to 8% of apparently healthy individuals [62] and have been studied in several other autoimmune diseases, infectious diseases and various kidney diseases [63, 64].

In SLE, several studies have associated anti-C1q with renal impairment caused by the disease [25, 65], a finding that has been reinforced by experimental studies demonstrating a possible pathogenic role of this autoantibody in SLE [66, 67].

Three SRs were included that evaluated the role of anti-C1q as a BM in LN [42, 45, 46]. Two of the SRs analysed the accuracy of anti-C1q for diagnosing LN among SLE patients and for detecting its activity [42, 46]. Yin et al. and Eggleton et al. showed partial overlap of the included studies. The review by Eggleton et al. encompassed all the articles that were included in the SR performed by Yin et al. and added 6 additional studies evaluating the accuracy of anti-C1q in the discrimination of patients with a current or previous history of LN [68–73]. In total, 32 studies were included: 28 studies (2769 patients) evaluated accuracy for diagnosing LN among patients with SLE, and 9 studies (249 patients) analysed diagnostic accuracy for monitoring LN activity. The 2 reviews showed results in the same direction, although Eggleton found overall accuracy measures higher than those found by Yin (**Table 2**), possibly because Eggleton included additional studies and used different statistical methods to summarize the results. The heterogeneity among the included studies was high in terms of the evaluation of this antibody’s accuracy for both the diagnosis of LN and the detection of its activity. No threshold effect was found in any of the analyses, and the covariates that were explored by meta-regression (quality of the study, detection method and ethnic group) did not influence the results. The Egger test, which was applied in the review by Yin, showed a significant probability of publication bias. Despite these limitations, anti-C1q was identified as a potential BM in LN.

The review by Wang et al. included only studies that were conducted in the Chinese population and evaluated the accuracy of anti-C1q in the diagnosis of LN in patients with SLE. A total of 11 studies were included corresponding to 1084 patients, among which 474 were diagnosed with LN. The pooled sensitivity was 0.67 (95% CI 0.63–0.71), the pooled specificity was 0.69 (95% CI 0.65–0.74), the positive likelihood ratio (PLR) was 2.18 (95% CI 1.75–2.72), the negative likelihood ratio (NLR) was 0.48 (95% CI 0.39–0.60), the diagnostic odds ratio (DOR) was 5.09 (3.29–7.85) and the SROC-AUC was 0.749. The heterogeneity among the studies was significant, with I^2^ values ranging from 43.6% for PLR to 88.9% for sensitivity. In the subgroup analysis of the possible sources of inconsistency, the methodological quality, the age of the evaluated population and the sample size were considered. However, none of these variables seemed to have a significant influence on heterogeneity, and no threshold effect was observed. Although the review included only studies of Chinese populations, the accuracy values, although lower, were not far from those found in the other 2 reviews, especially for PLR, NLR and DOR (**Table 2**). Despite the close publication dates of the SRs by Eggleton [42] and Wang [45], there was an intersection of only three primary studies. This reveals a deficit in the sensitivity of the search strategy used by the authors.

The role of anti-C1q as a BM in LN is not yet defined. In the SRs that were identified, it did not perform well for differentiating patients according to a positive or negative test. However, there seems to be a benefit to its use, which may have been obscured by the potential effect of the heterogeneity among the studies, and the sensitivity of the search strategy implemented by Eggleton.

#### Cytokines

*Tumour necrosis factor-like weak inducer of apoptosis (TWEAK)*. TWEAK is a proinflammatory cytokine in the TNF superfamily that activates fibroblast growth factor-inducible 14 (Fn14), a protein in the TNF receptor superfamily that is constitutively present in healthy tissues, and may increase its expression in inflammatory situations [74]. TWEAK is secreted mainly by monocytes and macrophages and participates in tissue repair and remodelling [74]. Several studies have indicated the involvement of the TWEAK-Fn14 axis in the pathogenesis of chronic autoimmune diseases, especially in cases of neurological, vascular and renal involvement [75].

Two systematic reviews focused on the diagnostic performance of TWEAK as a BM for lupus nephritis [43, 48]. The SR by Wang et al. [48] addressed the role of urinary TWEAK (uTWEAK) as a BM in LN, evaluating its diagnostic accuracy for detecting LN in patients with SLE and for monitoring LN activity. The analysis of the accuracy of TWEAK for the diagnosis of LN involved 4 studies (276 patients) and resulted in a pooled sensitivity of 0.55 (95% CI 0.47–0.63), a pooled specificity of 0.92 (95% CI 0.86–0,96), a DOR of 16.54 (95% CI 7.57–36.15) and an SROC-AUC of 0.822.

Regarding its diagnostic accuracy in the detection of nephritis activity, 3 studies were included. The pooled sensitivity was 0.91 (95% CI 0.82–0.96), the pooled specificity was 0.70 (95% CI 0.58–0.81), the DOR was 18.54 (7.45–45.87) and the SROC-AUC was 0.813. The SR by Wang et al. found low heterogeneity, opting for using a fixed-effects model for summary. Furthermore, the number of included studies in both arms of the review was small. No threshold effect was observed.

Ma et al. [43] reviewed primary studies assessing the diagnostic accuracy of serum and urinary TWEAK in predicting active LN in SLE patients. Nine studies were included (334 patients), 7 of which evaluated TWEAK in urine and 2 in serum (sTWEAK).

The summarized data involved both groups of studies evaluating serum and urinary TWEAK, and revealed a pooled sensitivity of 0,69 (95%CI 0,63–0,75), pooled specificity of 0,77 (95%CI 0,71–0,82), pooled positive likelihood ratio of 3,31 (95%CI 2,05–5,35), pooled negative likelihood ratio of 0,38 (95% CI 0,26–0,55), pooled DOR of 10,89 (95%CI 6,73–17,63) and a ROC/AUC of 0,827 (SE 0,0289). The heterogeneity found among the studies was moderate to high. The subgroup analysis revealed that the pooled sensitivity, DOR and AUC of TWEAK in predicting active LN were higher in patients with R-SLEDAI > 4 when compared to patients with R-SLEDAI > 0 (0,85 x 0,66; 19,00 x 8,90 and 0,90 x 0,79 respectively). uTWEAK also revealed a higher pooled DOR than sTWEAK (12,4 and 6,76, respectively). Moreover, TWEAK and R-SLEDAI were correlated in 6 of the included primary studies; and in 5 of them, the correlation was between TWEAK and proteinuria. There was no threshold effect.

Despite the review of Ma et al. being more recent and with a more significant number of studies, the overlapping of primary studies among both SRs comprised a total of 5 works [76–80], amounting to 6 the number of non-overlapping studies [81–86].

Regardless of the methodological differences between both SRs, the partial intersection of primary studies, and their heterogeneity, the results of both reviews point to uTWEAK as an auspicious BM for the clinical management of LN.

#### Chemokines

*Monocyte chemoattractant protein-1 (MCP-1)*. MCP-1 is a chemokine in the CC family that is composed of 76 amino acids and is produced by epithelial cells, endothelial cells, smooth muscle cells, monocytes, macrophages, fibroblasts, astrocytes and microglial cells under various stimuli, such as oxidative stress, cytokines and growth factors [87]. MCP-1 has been implicated in the pathogenesis of several diseases through its influence on chemotaxis and oxidative stress, among other actions [88–90]. In SLE, MCP-1 has been associated with disease activity and renal impairment [91, 92].

Only 1 SR was found on the use of MCP-1 as a BM in LN [47]. Xia et al. analysed primary studies, evaluating their diagnostic accuracy in detecting renal disease activity. Seven studies with a total of 521 participants were included. The pooled sensitivity was 0.89 (95% CI 0.86–0.93), the pooled specificity was 0.63 (95% CI 0.55–0.69), the PLR was 2.16 (95% CI 1.66–2.80), the NLR was 0.15 (95% CI 0.08–0.30), the DOR was 19.4 (95% CI 7.24–51.96) and the SROC-AUC was 0.90.

There was high heterogeneity among the studies, with an I^2^ of 75.4%. There was no threshold effect, and in the subgroup analysis, ethnicity and the proportion of inactive LN had no influence on the inconsistency that was observed. However, no sensitivity analysis was performed. There was no evidence of publication bias. Despite the limitations of the data, MCP-1 seems to be superior to the conventional serological BMs used in the management of LN.

*Interferon inducible protein-10 (IP-10)*. IP-10 or CXCL10 is a chemokine in the ELR-CXC family that is produced by T lymphocytes, natural killer (NK) cells, NK-T cells, neutrophils, monocytes, splenocytes, endothelial cells, fibroblasts, keratinocytes and other types of cells under the stimulus of proinflammatory cytokines [93]. It has chemotactic power over lymphocytes, participates in the regulation of cell growth and has angiostatic properties [94, 95]. The role of IP-10 has been studied in several autoimmune diseases, such as rheumatoid arthritis [96], Sjögren’s syndrome [97] and multiple sclerosis [98]. In SLE patients, studies have shown high levels of IP-10 in serum [99] and in samples from cutaneous lesions of the disease [100], and it appears to correlate with disease activity [101].

Puapatanakul et al. conducted a systematic review of studies that evaluated the serum and urinary levels of IP-10 in patients with SLE with and without LN. A total of 23 publications were included, and only 6 evaluated IP-10 specifically in LN. Most of the included studies did not evaluate diagnostic accuracy measures. The meta-analysis consisted of values that referred to mean differences between the studied groups. These showed no statistical significance of the serum IP-10 for differentiating between patients with LN and patients with SLE without nephritis, and only a tendency toward higher urinary concentrations in patients with LN than in patients without LN.

Only 2 studies evaluated the diagnostic accuracy of serum IP-10 levels for the detection of renal involvement in patients with SLE; however, no meta-analysis was performed. The studies presented an analysis of the area under the ROC curve (ROC-AUC), showing values ranging from 0.596 to 0.633, emphasizing the lack of utility of this BM for this outcome.

Among the studies that evaluated the urinary levels of IP-10 for the detection of renal involvement, only 5 studies analysed the ROC curve to demonstrate its overall performance. One of the studies (60 subjects) showed sensitivity 1,00, specificity 0,98, and an area under the ROC curve (ROC-AUC) of 1.000 [102]. In 3 other studies [81, 103, 104], the urinary IP-10 showed ROC-AUCs ranging from 0.595 to 0.680, which was not superior compared to the findings for conventional BMs.

In 1 of the included studies, urinary IP-10 levels were measured by mRNA detection by RT–PCR, and urinary IP-10 showed a good ability to distinguish Class IV LN (diffuse proliferative glomerulonephritis), with a sensitivity of 0.73, a specificity of 0.94 and an ROC-AUC of 0.89 (95% CI 0.78–0.99) [105]. However, the number of patients evaluated was small (26 subjects).

It was not possible to reach a conclusion regarding the diagnostic accuracy of IP-10 in LN. There was considerable disagreement among the diagnostic accuracy measures found in the various primary studies, the number of studies evaluating this aspect was small, and the population samples were also small. The SR of Puapatanakul found no difference between the mean serum levels of IP-10 in patients with active LN, those of patients with active SLE without LN and those of patients with inactive LN. Regarding urinary levels, only a statistical tendency was found for these to be higher in patients with nephritis; however, the heterogeneity among the studies was high. There was no report on subgroup analysis.

### Other molecules

*Neutrophil gelatinase-associated lipocalin (NGAL)*. NGAL is an acute phase glycoprotein belonging to the lipocalin family. Under conditions of homeostasis, it is secreted by neutrophils, macrophages, hepatocytes, adipocytes, neurons and epithelial cells, and its production is significantly increased under inflammatory stimulus, oxidative stress and tissue injury [106, 107]. Several studies have associated increased urinary NGAL concentrations with various types of kidney injury [108–110]. In SLE, an *in vitro* study by Qing et al. showed increased expression of Lipocalin-2 in mesangial cells derived from SLE patients after stimulation with anti-murine DNA antibody [111], and observational studies conducted in humans have shown higher urinary concentrations of NGAL in patients with LN [112, 113].

Two SRs evaluated the role of NGAL as a BM in LN [37, 47]. The review by Gao et al. is more recent (2020) and encompasses all of the primary studies evaluated by Fang et al. plus 8 additional articles, for a total of 19 articles. The evaluated outcomes by both reviews were the accuracy of uNGAL in the diagnosis of LN, the detection of LN activity, the prediction of LN relapse and the distinction between the proliferative and non-proliferative forms of LN (the latter outcome was evaluated only in the Gao review). The 2 SRs identified results in the same direction for the diagnostic accuracy of uNGAL. The most relevant results were for the diagnosis of LN in SLE patients. The summary measures reported by Gao (**Table 2**) are described next.

The 19 articles included in Gao et al. corresponded to 21 studies and a total of 1453 participants, including both adults (17 studies) and children (4 studies). The main method for the detection of uNGAL was ELISA, which was used in all primary studies except for 1 [114], which used a chemiluminescent microparticle (CMIA) immunoassay. The reference tests varied between the various studies and depending on the outcomes studied, as shown in **Table 2**.

Regarding the diagnosis of LN, data from 9 studies (573 subjects) [77, 112, 113, 115–120] were evaluated. The pooled sensitivity was 0.84 (95% CI 0.71–0.91), the pooled specificity was 0.91 (95% CI 0.70–0.98), the pooled PLR was 9.08 (95% CI 2.31–35.69), the pooled NLR was 0.18 (95% CI 0.09–0.35), the DOR was 50.51 (95% CI 8.15–313.03) and the area under the SROC curve (SROC-AUC) was 0.92 (95% CI 0.90–0.94).

Ten studies (949 subjects) [112, 115, 121–127] analysed the diagnostic accuracy for detecting kidney disease activity. The pooled sensitivity was 0.72 (95% CI 0.56–0.84), the pooled specificity was 0.71 (95% CI 0.51–0.84), the pooled PLR was 2.45 (95% CI 1.32–4.54), the pooled NLR was 0.39 (0.22–0.70), the DOR was 6.24 (95% CI 2.08–18.68) and the SROC-AUC was 0.77 (95% CI 0.74–0.81).

The diagnostic accuracy for predicting LN relapse was evaluated in 6 studies (442 subjects) [114, 122, 125, 127]. The pooled sensitivity was 0.80 (95% CI 0.57–0.92), the pooled specificity was 0.67 (95% CI 0.58–0.75), the pooled PLR was 2.41 (95% CI 1.57–3.72), the pooled NLR was 0.30 (95% CI 0.11–0.79), the DOR was 8.08 (95% CI 2.02–32.35) and the SROC-AUC was 0.74 (95% CI 0.70–0.78).

There was high heterogeneity among the studies for all outcomes evaluated, with I^2^ values ranging from 66.15% to 94.24%. In the meta-regression, subgroup and sensitivity analyses, a possible influence of the quality of the study (defined by the QUADAS-2 score) on accuracy for the diagnosis of LN among patients with SLE was identified. The higher-quality studies (QUADAS-2 ≥13) showed lower pooled sensitivity and higher pooled specificity than the lower-quality studies. The design of the study showed an influence on the results of the synthesis of accuracy for the detection of LN activity, with the cross-sectional studies showing higher pooled sensitivity and specificity values than the prospective cohort studies. The reference test that was used had an influence on accuracy for the prediction of relapses, with the studies that used R-SLEDAI showing higher pooled sensitivity and specificity and lower heterogeneity (a pooled sensitivity of 0.80 to 0.90, a pooled specificity of 0.67 to 0.74 and I^2^ values of 72.5% to 55.4% and 66.15% to 21.17%, respectively). However, the influence of the examined variables was partial, and other sources of influence were not identified. There was no threshold effect in any of the evaluated outcomes, and there was no evidence of publication bias.

### Methodological quality of the included reviews

**Fig 2:**
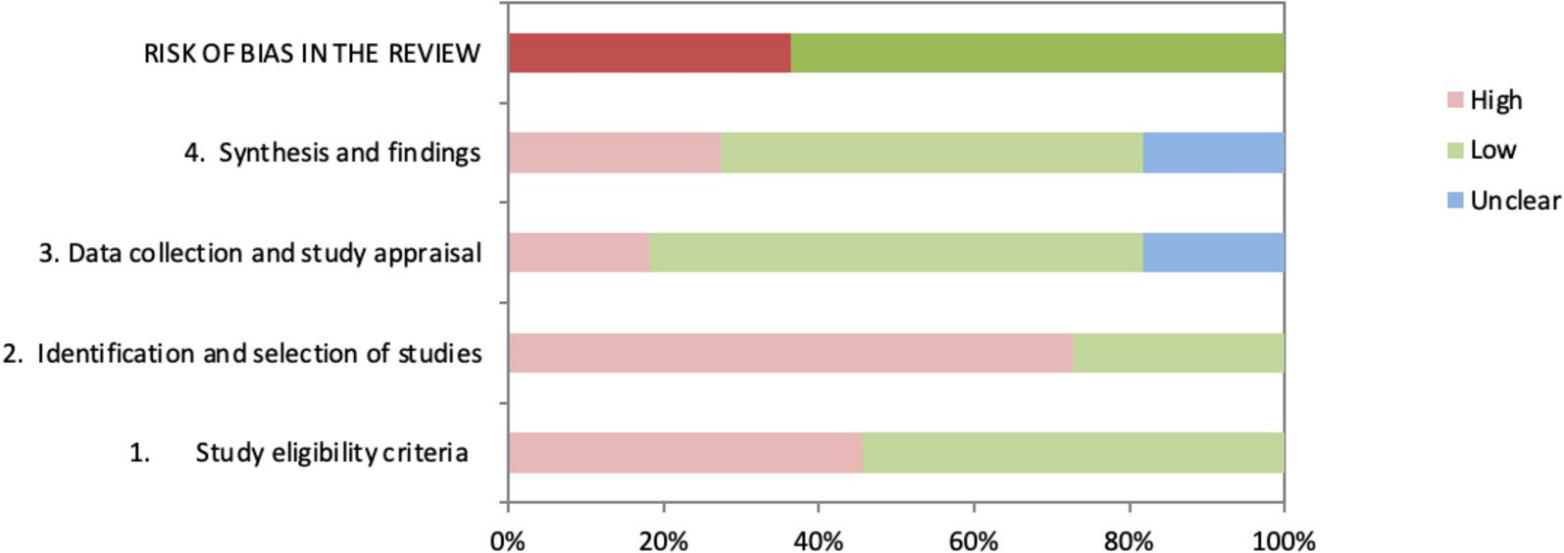
Risk of bias assessment with ROBIS tool. Darker colours indicate overall ROBIS rating; lighter colours concern judgments.

The results of the evaluation with the ROBIS tool showed that 7 of the 10 reviews had a low overall risk of bias. The included SRs presented their research questions in a way that was compatible with this overview. However, some were more comprehensive and did not have clearly defined PIRD components. The domains that most frequently presented risk of bias were those related to eligibility criteria and to the identification and selection of studies. None of the reviews reported the registration of a previous protocol, 5 presented restrictions of the inclusion of studies without justification (e.g., quality, language, date range etc.), 7 did not clearly report whether free or controlled terms were included in the search strategy, 9 did not include grey literature, and 2 did not report the use of at least two reviewers throughout the review process. All SRs used some tool to analyse the quality of the included primary studies or their risk of bias, and QUADAS and QUADAS-2 were the most frequently used tools. Most of the SRs considered the methodological quality and/or risk of bias of the included primary studies when interpreting the summarized results. The risk of bias of the included SRs, evaluated by the ROBIS tool, is shown graphically in **Fig 2 and Table 3**.

**Table 3:** Risk of bias assessment with ROBIS tool.

Review	Phase 2				Phase 3
	1. Study eligibility criteria	2. Identification and selection of studies	3. Data collection and study appraisal	4. Synthesis and findings	Risk of bias in the review
**Benito-Garcia 2004**	low risk	high risk	high risk	high risk	low risk
**Yin 2012**	high risk	high risk	low risk	low risk	low risk
**Eggleton 2014**	low risk	low risk	low risk	low risk	low risk
**Fang 2015**	low risk	low risk	?	low risk	low risk
**Wang 2015**	low risk	high risk	low risk	low risk	high risk
**Puapatanakul 2019**	high risk	high risk	?	high risk	high risk
**Gao 2020**	low risk	low risk	low risk	low risk	low risk
**Wang 2020**	high risk	high risk	low risk	low risk	low risk
**Xia 2020**	high risk	high risk	low risk	?	high risk
**Ma 2021**	low risk	high risk	low risk	?	low risk

? = unclear risk

## Discussion

LN is one of the most relevant impairments in SLE because it has a significant prevalence among patients (30 to 60%) and a great impact on prognosis. Regardless of advances in treatment, approximately 10% of patients still progress to end-stage renal disease in the first 5 years after diagnosis and have a risk of death 8 times higher than that of the general population [128].

Although the term “lupus nephritis” gives the impression of a single type of lesion, it comprises a diverse set of kidney injuries that can compromise any of the tissue compartments of the kidney with varying degrees of association; this results in clinical manifestations of variable severity and evolution [129], which makes the discovery of good BMs a great challenge.

This overview found 10 SRs that addressed the diagnostic accuracy of new serum and urinary BMs in LN. Among these, the following BMs were evaluated: (a) antibodies (anti-Sm, anti-RNP and anti-C1q) [41, 42, 45, 46], (b) cytokines (TWEAK and MCP-1) [43, 48, 49], (c) a chemokine (IP-10) [39] and (d) an acute phase glycoprotein (NGAL) [37, 47], as previously described.

The SRs identified mainly primary studies that answered questions about the accuracy of the BM for the diagnosis of LN in patients with SLE and for the detection of LN activity. Only the 2 SRs on uNGAL [37, 47] also evaluated studies regarding the accuracy of the BMs for predicting LN relapse, and only the review of Gao et al. analysed studies of the accuracy of a BM (NGAL) for distinguishing the histopathological type (proliferative and non-proliferative LN) [37].

Anti-Sm and anti-RNP showed to be of no use in the detection of LN [41]. Although the SR by Benito-Garcia et al. included primary studies that were of good methodological quality and that evaluated a significant number of individuals (984 for anti-Sm and 1114 for anti-RNP), its search strategy was restricted to studies reported in English and included only 2 databases, which overlap (PubMed and Medline). This confers a reasonable risk that relevant studies were not included. In addition, the confidence intervals for the summarized sensitivity and specificity values were wide. Thus, despite the possibility that these antibodies are not useful as BMs in LN, a more sensitive search would provide a definitive answer regarding their role in this type of SLE impairment.

One SR that examined IP-10 was found [39], and it evaluated studies that considered serum and urinary levels of this BM. Five studies were included in the review arm that evaluated serum IP-10. However, only 2 studies performed ROC curve analyses (without meta-analysis), and those showed a poor performance of the BM for detecting nephritis among patients with SLE. Additionally, the meta-analysis of the mean differences (MD) between patients with active LN and patients with SLE without nephritis in the 5 included studies showed no difference. This difference was only significant when patients were compared with healthy controls (as in 3 of the studies).

On the other hand, of the 6 included studies that evaluated urinary IP-10, 5 reported accuracy data with ROC curve analyses. The results were varied but pointed in the same direction, indicating a probable benefit of urinary IP-10 as a BM. However, the review did not provide a quantitative synthesis of these results. The authors metanalysed 3 studies that evaluated mean differences, comparing patients with lupus nephritis, patients with inactive SLE, and patients with active SLE without nephritis. The summary mean showed only a tendency for the mean urinary levels of IP-10 to be higher among patients with nephritis. Thus, although the results for serum IP-10 are not encouraging, urinary IP-10 seems to have relevance as a BM in LN and is deserving of further primary studies.

The BMs with the best accuracy profile were uMCP-1, uTWEAK, uNGAL and anti-C1q, which were more sensitive than specific, in most occasions, for the analysed outcomes [37, 42, 43, 45–49]. The best sensitivity values were found for the accuracy of detection of nephritis activity. This finding may have been favoured by the fact that these studies compared clearly inflamed subjects (those with active LN) with groups of individuals with clinically inactive disease (with no or little inflammation). This made the composition of each group more homogeneous and, clinically, more distinct from each other, which tended to increase the differences between them.

The sensitivity of a BM varies not only according to test cut-off used but according to the severity of the disease [130]. In the context of LN, other factors, such as the affected renal compartments (mesangial, interstitial, vascular, glomerular or tubules), the predominant location of the immune complex deposit (subendothelial or subepithelial), the type of pathological lesion (proliferative or non proliferative) and the established degree of chronicity, are also likely to influence the performance of accuracy measures of the BM being tested.

Thus, an important consideration in the study of BMs in the context of LN is the stratification of patients by (a) the presence of disease activity, (b) clinical severity, (c) histopathological features, (d) the mean time of kidney disease and (e) treatment. This would require a large population sample, which may be more feasible for multicentre research collaborations, and the standardization of smaller studies in terms of the details of the research design used to evaluate diagnostic accuracy in LN. Such efforts could facilitate the subsequent summarization of results and accelerate progress in this area of knowledge.

In this overview, the SRs that were included did not explore in depth the composition of each comparison group within the primary studies. The proportion of individuals with active disease and the histopathological class of nephritis were not discussed in most of the included reviews, and some reviews did not explore the age of the participants. These variables may have significantly influenced the heterogeneity of the summarized results.

Another relevant issue was the design of the primary studies included in the SRs. Many diagnostic accuracy studies have a cross-sectional design, which may overestimate or understimate the findings when there are individuals in the sample with the disease in different clinical stages or when the reference test is not 100% accurate [131]. In the SR of Gao [37], the sensitivity of uNGAL in the arm of the review that evaluated its accuracy for the detection of was 0.72 (95%CI 0.56–0.84). During the analysis of heterogeneity, it was observed that the cohort studies decreased the pooled sensitivity compared to the cross-sectional studies (0.87 x 0.57).

Renal biopsy (the gold standard for diagnosis) is not repeated regularly as a matter of clinical routine because of its invasive and risky nature. Instead, the detection of renal impairment relies on laboratory tests and activity scoring tools (e.g., R-SLEDAI). This restricts the evaluation of new BMs because their accuracy may be underestimated or overestimated due to the limitations of the reference tests. Cohort studies would most likely generate accuracy measures closer to reality in this context, because they allow the programmed collection of biological material for the index biomarker estimation before clinical manifestations or the positivity of the reference test. Moreover, it would grant posterior diagnostic confirmation during the follow-up.

Thus, cohort studies with pre-programmed biological material collection would allow a correct evaluation of the accuracy of the index test, as it would be assessed at various times until evident kidney disease occurs. Among the SRs included in this overview, only 3 reported the design of the included primary studies, which made it difficult to interpret the totality of summarized data.

Despite 7 of the 10 included SRs being from China, only 1 SR [45] analyzed primary studies restricted to the Chinese population. Eight SRs had no racial restrictions and included studies in populations from North and South Americas, Europe, Asia, and Africa with a heterogeneous ethnic composition. Two SRs [46, 49] included ethnic background on subgroup analysis and did not find significant interference.

Another relevant point is the use of BM panels. The histopathological and pathophysiological diversity of LN requires a set of BMs that reflect the various phenomena in progress within renal tissue. Despite the significant heterogeneity of the results summarized in the included SRs and the limitations that are already known as a result of accuracy studies, the data found in this overview highlight urinary MCP-1, TWEAK, NGAL and anti-C1q as useful BMs in LN, and the inclusion of these in a diagnostic panel offers a promising research approach with existing initiatives [132–136].

This is the first overview to synthesize the existing evidence reported by SRs of the diagnostic accuracy of new serum and urinary BMs in LN. With more than 30 BMs undergoing research in this field and the ongoing discovery of new potential BMs, the synthesis of the existing evidence provides an objective view of the direction of the data on studied BMs and unveils the best paths to be followed in related research.

Our overview had a wide scope, including 6 databases, grey literature and no time range or language restrictions. However, despite the advantage of providing a panoramic and objective view of the existing evidence on a subject, the results of an overview are subject to failures arising from the handling of secondary data. In our overview, some SRs restricted their search to the English language, used few databases and did not include grey literature, which may have led to the loss of relevant studies.

In addition, none of the SRs had previously registered their protocols, and some did not report the involvement of at least 2 reviewers in all phases of the review, which increases the chance of errors and ad hoc changes that can lead to spurious results. In addition, the heterogeneity among the primary studies, a common problem of SRs and overviews, as well as the variability in the statistical methods used to summarize the data among the SRs, requires careful interpretation.

## Conclusion

Our results show that in spite of the numerous biomarkers being studied for LN, there are only a few BMs responsible for most primary studies, with 10 SRs analysing their diagnostic accuracy. They highlight that anti-C1q, urinary MCP-1, TWEAK and NGAL deserve additional research attention, preferably with standardized methods and composing LN diagnostic panels in cohort studies and clinical diagnostic randomised trials, to obtain a better understanding of their usefulness and possibly validate their clinical use in the future.

## Supporting information

S1 Checklist PRISMA 2020 checklist article LN BM 200422. (PDF)

S1 Table Data bases and search strategies used. (DOCX)

S2 Table Overview of reported cut-off thresholds used on primary studies in the included SRs. (PDF)

## References

[1] Lee YH, Choi SJ, Ji JD, Song GG. Overall and cause-specific mortality in systemic lupus erythematosus: an updated meta-analysis Lupus. 2016;25(7):727–34. 26811368 10.1177/0961203315627202

[2] Anders HJ, Saxena R, Zhao MH, Parodis I, Salmon JE, Mohan C. Lupus nephritis. Nat Rev Dis Primers. 2020;6(1):7. doi: 10.1038/s41572-019-0141-9 31974366

[3] Cervera R, Khamashta MA, Font J, Sebastiani GD, Gil A, Lavilla P, et al. Morbidity and mortality in systemic lupus erythematosus during a 10-year period: a comparison of early and late manifestations in a cohort of 1,000 patients. Medicine (Baltimore). 2003;82(5):299–308. doi: 10.1097/01.md.0000091181.93122.55 14530779

[4] Mok CC, To CH, Ho LY, Yu KL. Incidence and Mortality of Systemic Lupus Erythematosus in a Southern Chinese Population, 2000–2006. J Rheumatol. 2008;35(10):1978–82. 18688913

[5] Houssiau FA, Vasconcelos C, D’Cruz D, Sebastiani GD, de Ramon Garrido E, Danieli MG, et al. The 10-year follow-up data of the Euro-Lupus Nephritis Trial comparing low-dose and high-dose intravenous cyclophosphamide. Ann Rheum Dis. 2010;69(1):61–4. doi: 10.1136/ard.2008.102533 19155235

[6] Houssiau FA, Vasconcelos C, D’Cruz D, Sebastiani GD, de Ramon Garrido E, Danieli MG, et al. Early response to immunosuppressive therapy predicts good renal outcome in lupus nephritis: lessons from long-term followup of patients in the Euro-Lupus Nephritis Trial. Arthritis Rheum. 2004;50(12):3934–40. doi: 10.1002/art.20666 15593207

[7] Moroni G, Radice A, Giammarresi G, Quaglini S, Gallelli B, Leoni A, et al. Are laboratory tests useful for monitoring the activity of lupus nephritis? A 6-year prospective study in a cohort of 228 patients with lupus nephritis. Ann Rheum Dis. 2009;68(2):234–7. doi: 10.1136/ard.2008.094508 18718989

[8] Wang H, Ren YL, Chang J, Gu L, Sun LY. A Systematic Review and Meta-analysis of Prevalence of Biopsy-Proven Lupus Nephritis. Arch Rheumatol. 2018;33(1):17–25. doi: 10.5606/ArchRheumatol.2017.6127 29900975 PMC5864167

[9] Tektonidou MG, Dasgupta A, Ward MM. Risk of End-Stage Renal Disease in Patients With Lupus Nephritis, 1971–2015: A Systematic Review and Bayesian Meta-Analysis. Arthritis Rheumatol. 2016;68(6):1432–41. doi: 10.1002/art.39594 26815601 PMC5071782

[10] Gasparotto M, Gatto M, Binda V, Doria A, Moroni G. Lupus nephritis: clinical presentations and outcomes in the 21st century. Rheumatology (Oxford). 2020;59(Suppl5):v39–v51. doi: 10.1093/rheumatology/keaa381 33280015 PMC7751166

[11] Umeda R, Ogata S, Hara S, Takahashi K, Inaguma D, Hasegawa M, et al. Comparison of the 2018 and 2003 International Society of Nephrology/Renal Pathology Society classification in terms of renal prognosis in patients of lupus nephritis: a retrospective cohort study. Arthritis Res Ther. 2020;22(1):260. doi: 10.1186/s13075-020-02358-x 33148339 PMC7640657

[12] Weening JJ, D’Agati VD, Schwartz MM, Seshan SV, Alpers CE, Appel GB, et al. The classification of glomerulonephritis in systemic lupus erythematosus revisited. J Am Soc Nephrol. 2004;15(2):241–50. doi: 10.1097/01.asn.0000108969.21691.5d 14747370

[13] Bajema IM, Wilhelmus S, Alpers CE, Bruijn JA, Colvin RB, Cook HT, et al. Revision of the ISN/RPS classification for lupus nephritis: modified NIH activity and chronicity indices and clarification of definitions. Kidney International. 2018;93(4):789–96. 29459092 10.1016/j.kint.2017.11.023

[14] Corapi KM, Chen JL, Balk EM, Gordon CE. Bleeding complications of native kidney biopsy: a systematic review and meta-analysis. Am J Kidney Dis. 2012;60(1):62–73. doi: 10.1053/j.ajkd.2012.02.330 22537423

[15] Tunnicliffe DJ, Singh-Grewal D, Kim S, Craig JC, Tong A. Diagnosis, Monitoring, and Treatment of Systemic Lupus Erythematosus: A Systematic Review of Clinical Practice Guidelines. Arthritis Care Res (Hoboken). 2015;67(10):1440–52. doi: 10.1002/acr.22591 25778500

[16] Wilhelmus S, Bajema IM, Bertsias GK, Boumpas DT, Gordon C, Lightstone L, et al. Lupus nephritis management guidelines compared. Nephrol Dial Transplant. 2016;31(6):904–13. doi: 10.1093/ndt/gfv102 25920920

[17] Esdaile JM, Joseph L, Abrahamowicz M, Li Y, Danoff D, Clarke AE. Routine immunologic tests in systemic lupus erythematosus: is there a need for more studies? J Rheumatol. 1996;23(11):1891–6. 8923362

[18] Malvar A, Pirruccio P, Alberton V, Lococo B, Recalde C, Fazini B, et al. Histologic versus clinical remission in proliferative lupus nephritis. Nephrol Dial Transplant. 2017;32(8):1338–44. doi: 10.1093/ndt/gfv296 26250434 PMC5837387

[19] Maroz N, Segal MS. Lupus nephritis and end-stage kidney disease. Am J Med Sci. 2013;346(4):319–23. doi: 10.1097/MAJ.0b013e31827f4ee3 23370533

[20] Aguirre-Valencia D, Rios-Serna LJ, Posso-Osorio I, Naranjo-Escobar J, Lopez D, Bedoya-Joaqui V, et al. Expression of BAFF, APRIL, and cognate receptor genes in lupus nephritis and potential use as urinary biomarkers. J Transl Autoimmun. 2020;3:100027. doi: 10.1016/j.jtauto.2019.100027 32743512 PMC7388398

[21] Lu J, Kwan BC, Lai FM, Choi PC, Tam LS, Li EK, et al. Gene expression of TWEAK/Fn14 and IP-10/CXCR3 in glomerulus and tubulointerstitium of patients with lupus nephritis. Nephrology (Carlton). 2011;16(4):426–32. doi: 10.1111/j.1440-1797.2011.01449.x 21303425

[22] Wither JE, Prokopec SD, Noamani B, Chang NH, Bonilla D, Touma Z, et al. Identification of a neutrophil-related gene expression signature that is enriched in adult systemic lupus erythematosus patients with active nephritis: Clinical/pathologic associations and etiologic mechanisms. PLoS One. 2018;13(5):e0196117. doi: 10.1371/journal.pone.0196117 29742110 PMC5942792

[23] Yang H, Li H. CD36 identified by weighted gene co-expression network analysis as a hub candidate gene in lupus nephritis. PeerJ. 2019;7:e7722. doi: 10.7717/peerj.7722 31592160 PMC6777479

[24] Ben-Ami Shor D, Blank M, Reuter S, Matthias T, Beiglass I, Volkov A, et al. Anti-ribosomal-P antibodies accelerate lupus glomerulonephritis and induce lupus nephritis in naive mice. J Autoimmun. 2014;54:118–26. 24662148 10.1016/j.jaut.2014.02.013

[25] Metwally IM, Eesa NN, Yacoub MH, Elsman RM. Association of anti-nuclesome and anti C1q antibodies with lupus nephritis in an Egyptian cohort of patients with systemic lupus erythematosus. Adv Rheumatol. 2019;59(1):10. doi: 10.1186/s42358-019-0054-z 30832710

[26] Zhang WH, Pan HF, Zhao XF, Ye DQ, Li XP, Xu JH. Anti-alpha-actinin antibodies in relation to new-onset systemic lupus erythematosus and lupus nephritis. Mol Biol Rep. 2010;37(3):1341–5. doi: 10.1007/s11033-009-9513-7 19319662

[27] Jakiela B, Kosałka J, Plutecka H, Węgrzyn AS, Bazan-Socha S, Sanak M, et al. Urinary cytokines and mRNA expression as biomarkers of disease activity in lupus nephritis. Lupus. 2018;27(8):1259–70. doi: 10.1177/0961203318770006 29653499

[28] Selvaraja M, Abdullah M, Arip M, Chin VK, Shah A, Amin Nordin S. Elevated interleukin-25 and its association to Th2 cytokines in systemic lupus erythematosus with lupus nephritis. PLoS One. 2019;14(11):e0224707. doi: 10.1371/journal.pone.0224707 31697750 PMC6837487

[29] Daniel L, Sichez H, Giorgi R, Dussol B, Figarella-Branger D, Pellissier JF, et al. Tubular lesions and tubular cell adhesion molecules for the prognosis of lupus nephritis. Kidney Int. 2001;60(6):2215–21. doi: 10.1046/j.1523-1755.2001.00055.x 11737595

[30] Nakatani K, Fujii H, Hasegawa H, Terada M, Arita N, Ito MR, et al. Endothelial adhesion molecules in glomerular lesions: association with their severity and diversity in lupus models. Kidney Int. 2004;65(4):1290–300. doi: 10.1111/j.1523-1755.2004.00537.x 15086468

[31] Skeoch S, Haque S, Pemberton P, Bruce IN. Cell adhesion molecules as potential biomarkers of nephritis, damage and accelerated atherosclerosis in patients with SLE. Lupus. 2014;23(8):819–24. doi: 10.1177/0961203314528061 24647443 PMC4232262

[32] Mohammed JA, Mok AY, Parbtani A, Matsell DG. Increased expression of insulin-like growth factors in progressive glomerulonephritis of the MRL lpr mouse. Lupus. 2003;12(8):584–90. doi: 10.1191/0961203303lu422oa 12945716

[33] Resende AL, Elias RM, Wolf M, Dos Reis LM, Graciolli FG, Santos GD, et al. Serum levels of fibroblast growth factor 23 are elevated in patients with active Lupus nephritis. Cytokine. 2017;91:124–7. doi: 10.1016/j.cyto.2016.12.022 28063327

[34] Bellan M, Quaglia M, Nerviani A, Mauro D, Lewis M, Goegan F, et al. Increased plasma levels of Gas6 and its soluble tyrosine kinase receptors Mer and Axl are associated with immunological activity and severity of lupus nephritis. Clin Exp Rheumatol. 2021;39(1):132–8. doi: 10.55563/clinexprheumatol/xyylza 32573415

[35] Liu P, Li P, Peng Z, Xiang Y, Xia C, Wu J, et al. Predictive value of the neutrophil-to-lymphocyte ratio, monocyte-to-lymphocyte ratio, platelet-to-neutrophil ratio, and neutrophil-to-monocyte ratio in lupus nephritis. Lupus. 2020;29(9):1031–9. doi: 10.1177/0961203320929753 32501169

[36] Yap DYH, Yung S, Lee P, Yam IYL, Tam C, Tang C, et al. B Cell Subsets and Cellular Signatures and Disease Relapse in Lupus Nephritis. Front Immunol. 2020;11:1732. doi: 10.3389/fimmu.2020.01732 33013825 PMC7511550

[37] Gao Y, Wang B, Cao J, Feng S, Liu B. Elevated Urinary Neutrophil Gelatinase-Associated Lipocalin Is a Biomarker for Lupus Nephritis: A Systematic Review and Meta-Analysis. Biomed Res Int. 2020;2020:2768326. doi: 10.1155/2020/2768326 32685458 PMC7346103

[38] Lee YH, Song GG. Urinary MCP-1 as a biomarker for lupus nephritis: a meta-analysis. Z Rheumatol. 2017;76(4):357–63. 27278779 10.1007/s00393-016-0109-z

[39] Puapatanakul P, Chansritrakul S, Susantitaphong P, Ueaphongsukkit T, Eiam-Ong S, Praditpornsilpa K, et al. Interferon-Inducible Protein 10 and Disease Activity in Systemic Lupus Erythematosus and Lupus Nephritis: A Systematic Review and Meta-Analysis. Int J Mol Sci. 2019;20(19). doi: 10.3390/ijms20194954 31597273 PMC6801540

[40] Whiting P, Savovic J, Higgins JP, Caldwell DM, Reeves BC, Shea B, et al. ROBIS: A new tool to assess risk of bias in systematic reviews was developed. J Clin Epidemiol. 2016;69:225–34. doi: 10.1016/j.jclinepi.2015.06.005 26092286 PMC4687950

[41] Benito-Garcia E, Schur PH, Lahita R, American College of Rheumatology Ad Hoc Committee on Immunologic Testing G. Guidelines for immunologic laboratory testing in the rheumatic diseases: anti-Sm and anti-RNP antibody tests. Arthritis Rheum. 2004;51(6):1030–44. 15593352 10.1002/art.20836

[42] Eggleton P, Ukoumunne OC, Cottrell I, Khan A, Maqsood S, Thornes J, et al. Autoantibodies against C1q as a Diagnostic Measure of Lupus Nephritis: Systematic Review and Meta-analysis. J Clin Cell Immunol. 2014;5(2):210. doi: 10.4172/2155-9899.1000210 24955287 PMC4062947

[43] Ma HY, Chen S, Cao WD, Min CT. Diagnostic value of TWEAK for predicting active lupus nephritis in patients with systemic lupus erythematosus: a systematic review and meta-analysis. Renal Failure. 2021;43(1):20–31. doi: 10.1080/0886022X.2020.1853568 33307926 PMC7745842

[44] Radin M, Miraglia P, Barinotti A, Fenoglio R, Roccatello D, Sciascia S. Prognostic and Diagnostic Values of Novel Serum and Urine Biomarkers in Lupus Nephritis: A Systematic Review. American Journal of Nephrology. 2021;52(7):559–71. doi: 10.1159/000517852 34515043

[45] Wang ZX, Wang QX, Zeng HB, Shi J. Diagnostic value of serum anti-C1q antibodies for lupus nephritis in chinese population: A meta-analysis. Chinese Journal of Evidence-Based Medicine. 2015;15(8):914–21.

[46] Yin Y, Wu X, Shan G, Zhang X. Diagnostic value of serum anti-C1q antibodies in patients with lupus nephritis: A meta-analysis. Lupus. 2012;21(10):1088–97. doi: 10.1177/0961203312451202 22777943

[47] Fang YG, Chen NN, Cheng YB, Sun SJ, Li HX, Sun F, et al. Urinary neutrophil gelatinase-associated lipocalin for diagnosis and estimating activity in lupus nephritis: A meta-analysis. Lupus. 2015;24(14):1529–39. doi: 10.1177/0961203315600244 26314302

[48] Wang ZH, Dai ZW, Dong YY, Wang H, Yuan FF, Wang B, et al. Urinary Tumor Necrosis Factor-Like Weak Inducer of Apoptosis as a Biomarker for Diagnosis and Evaluating Activity in Lupus Nephritis: A Meta-analysis. Journal of clinical rheumatology: practical reports on rheumatic & musculoskeletal diseases. 2020. 10.1097/RHU.0000000000001316 32028305

[49] Xia YR, Li QR, Wang JP, Guo HS, Bao YQ, Mao YM, et al. Diagnostic value of urinary monocyte chemoattractant protein-1 in evaluating the activity of lupus nephritis: a meta-analysis. Lupus. 2020;29(6):599–606. doi: 10.1177/0961203320914372 32208799

[50] Migliorini P, Baldini C, Rocchi V, Bombardieri S. Anti-Sm and anti-RNP antibodies. Autoimmunity. 2005;38(1):47–54. doi: 10.1080/08916930400022715 15804705

[51] Aringer M, Costenbader K, Daikh D, Brinks R, Mosca M, Ramsey-Goldman R, et al. 2019 European League Against Rheumatism/American College of Rheumatology Classification Criteria for Systemic Lupus Erythematosus. Arthritis Rheumatol. 2019;71(9):1400–12. doi: 10.1002/art.40930 31385462 PMC6827566

[52] Ahn SS, Yoo BW, Song JJ, Park YB, Lee SK, Lee SW. Anti-Sm is associated with the early poor outcome of lupus nephritis. International Journal of Rheumatic Diseases. 2016;19:897–902. doi: 10.1111/1756-185X.12880 27126359

[53] Hirohata S, Sakuma Y, Yanagida T, Yoshio T. Association of cerebrospinal fluid anti-Sm antibodies with acute confusional state in systemic lupus erythematosus. Arthritis Research & Therapy. 2014;16:450. doi: 10.1186/s13075-014-0450-z 25273532 PMC4203882

[54] Ishizaki J, Saito K, Nawata M, Mizuno Y, Tokunaga M, Sawamukai N, et al. Low complements and high titre of anti-Sm antibody as predictors of histopathologically proven silent lupus nephritis without abnormal urinalysis in patients with systemic lupus erythematosus. Rheumatology (Oxford). 2015;54(3):405–12. doi: 10.1093/rheumatology/keu343 25183834

[55] Ryu S, Fu W, Petri MA. Associates and predictors of pleurisy or pericarditis in SLE. Lupus Sci Med. 2017;4(1):e000221. doi: 10.1136/lupus-2017-000221 29118999 PMC5663266

[56] Barada FA, Andrews BS, Davis JS, Taylor RP. Antibodies to sm in patients with systemic lupus erythematosus. Arthritis and Rheumatism. 1981;24(10):1236–44. 6975630 10.1002/art.1780241003

[57] Field M, Williams DG, Charles P, Maini RN. Specificity of anti-Sm antibodies by ELISA for Systemic lupus erythematosus: increased sensitivity of detection using purified peptide antigens. Annals of the Rheumatic diseases. 1988;47:820–25. doi: 10.1136/ard.47.10.820 3143318 PMC1003610

[58] Janwityanuchit S, Verasertniyom O, Vanichapuntu M, Vatanasuk M. Anti-Sm: Its Predictive Value in Systemic Lupus Erythematosus. Clinical Rheumatology. 1993;12(3):350–53. doi: 10.1007/BF02231577 8258234

[59] McCain GA, Bell DA, Chodirker WB, Komar RR. Antibody to extractable nuclear antigen in the rheumatic diseases. J Rheumatol. 1978;5(4):399–406. 310883

[60] Winn DM, Wolfe JF, Lindberg DA, Fristoe FH, Kingsland L, Sharp GC. Identification of a clinical subset of systemic lupus erythematosus by antibodies to the Sm antigen. Arthritis and Rheumatism. 1979;22(12):1334–37. doi: 10.1002/art.1780221203 391237

[61] Jaeschke R, Guyatt GH, Sackett DL, Guyatt G, Bass E, Brill-Edwards P, et al. Users’ Guides to the Medical Literature: III. How to Use an Article About a Diagnostic Test B. What Are the Results and Will They Help Me in Caring for My Patients? JAMA. 1994;271(9):703–7. 8309035 10.1001/jama.271.9.703

[62] Potlukova E, Jiskra J, Limanova Z, Kralikova P, Smutek D, Mareckova H, et al. Autoantibodies against complement C1q correlate with the thyroid function in patients with autoimmune thyroid disease. Clin Exp Immunol. 2008;153(1):96–101. doi: 10.1111/j.1365-2249.2008.03670.x 18505435 PMC2432103

[63] Koma T, Veljkovic V, Anderson DE, Wang LF, Rossi SL, Shan C, et al. Zika virus infection elicits auto-antibodies to C1q. Sci Rep. 2018;8(1):1882. doi: 10.1038/s41598-018-20185-8 29382894 PMC5789871

[64] Orbai AM, Truedsson L, Sturfelt G, Nived O, Fang H, Alarcon GS, et al. Anti-C1q antibodies in systemic lupus erythematosus. Lupus. 2015;24(1):42–9. doi: 10.1177/0961203314547791 25124676 PMC4268323

[65] Kianmehr N, Khoshmirsafa M, Shekarabi M, Falak R, Haghighi A, Masoodian M, et al. High frequency of concurrent anti-C1q and anti-dsDNA but not anti-C3b antibodies in patients with Lupus Nephritis. J Immunoassay Immunochem. 2021;42(4):406–23. doi: 10.1080/15321819.2021.1895215 33788670

[66] Sun J, Guo S, Niu F, Liu D, Zhuang Y. Complement 1q protects MRL/lpr mice against lupus nephritis via inhibiting the nuclear factor-κB pathway. Mol Med Rep. 2020;22(6):5436–43. doi: 10.3892/mmr.2020.11588 33173997

[67] Wu WJ, Tan Y, Liu XL, Yu F, Zhao MH. C1q A08 Is a Half-Cryptic Epitope of Anti-C1q A08 Antibodies in Lupus Nephritis and Important for the Activation of Complement Classical Pathway. Front Immunol. 2020;11:848. doi: 10.3389/fimmu.2020.00848 32536911 PMC7267003

[68] Cai X, Yang X, Lian F, Lin X, Liang M, Li J, et al. Correlation between serum anti-C1q antibody levels and renal pathological characteristics and prognostic significance of anti-C1q antibody in lupus nephritis. J Rheumatol. 2010;37(4):759–65. doi: 10.3899/jrheum.090779 20194446

[69] Coremans IE, Spronk PE, Bootsma H, Daha MR, van der Voort EA, Kater L, et al. Changes in antibodies to C1q predict renal relapses in systemic lupus erythematosus. Am J Kidney Dis. 1995;26(4):595–601. doi: 10.1016/0272-6386(95)90595-2 7573013

[70] Jesus AA, Campos LM, Liphaus BL, Carneiro-Sampaio M, Mangueira CL, Rosseto EA, et al. Anti-C1q, anti-chromatin/nucleosome, and anti-dsDNA antibodies in juvenile systemic lupus erythematosus patients. Rev Bras Reumatol. 2012;52(6):976–81. 23223707

[71] Pradhan V, Patwardhan M, Nadkarni A, Ghosh K. Fc γ RIIA Genotypes and Its Association with Anti-C1q Autoantibodies in Lupus Nephritis (LN) Patients from Western India. Autoimmune Dis. 2010;2010:470695. doi: 10.4061/2010/470695 21188236 PMC3005808

[72] Pradhan V, Rajadhyaksha A, Mahant G, Surve P, Patwardhan M, Dighe S, et al. Anti-C1q antibodies and their association with complement components in Indian systemic lupus erythematosus patients. Indian J Nephrol. 2012;22(5):353–7. doi: 10.4103/0971-4065.103911 23326045 PMC3544056

[73] Ravelli A, Wisnieski JJ, Ramenghi B, Ballardini G, Zonta L, Martini A. IgG autoantibodies to complement C1q in pediatric-onset systemic lupus erythematosus. Clin Exp Rheumatol. 1997;15(2):215–9. 9196878

[74] Wajant H. The TWEAK-Fn14 system as a potential drug target. Br J Pharmacol. 2013;170(4):748–64. doi: 10.1111/bph.12337 23957828 PMC3799590

[75] Xu WD, Zhao Y, Liu Y. Role of the TWEAK/Fn14 pathway in autoimmune diseases. Immunol Res. 2016;64(1):44–50. doi: 10.1007/s12026-015-8761-y 26659091

[76] Dong X, Zheng Z, Luo X, Ding J, Li Y, Li Z, et al. Combined utilization of untimed single urine of MCP-1 and TWEAK as a potential indicator for proteinuria in lupus nephritis: A case-control study. Medicine (Baltimore). 2018;97(16):e0343. doi: 10.1097/MD.0000000000010343 29668584 PMC5916697

[77] El Shahawy MS, Hemida MH, Abdel-Hafez HA, El-Baz TZ, Lotfy AM, Emran TM. Urinary neutrophil gelatinase-associated lipocalin as a marker for disease activity in lupus nephritis. Scand J Clin Lab Invest. 2018;78(4):264–8. doi: 10.1080/00365513.2018.1449242 29533691

[78] Reyes-Martínez F, Pérez-Navarro M, Rodríguez-Matías A, Soto-Abraham V, Gutierrez-Reyes G, Medina-Avila Z, et al. Assessment of urinary TWEAK levels in Mexican patients with untreated lupus nephritis: An exploratory study. Nefrologia (Engl Ed). 2018;38(2):152–60. doi: 10.1016/j.nefro.2017.04.005 28755900

[79] Salem MN, Taha HA, Abd El-Fattah El-Feqi M, Eesa NN, Mohamed RA. Urinary TNF-like weak inducer of apoptosis (TWEAK) as a biomarker of lupus nephritis. Z Rheumatol. 2018;77(1):71–7. 27619369 10.1007/s00393-016-0184-1

[80] Schwartz N, Rubinstein T, Burkly LC, Collins CE, Blanco I, Su L, et al. Urinary TWEAK as a biomarker of lupus nephritis: a multicenter cohort study. Arthritis Res Ther. 2009;11(5):R143. doi: 10.1186/ar2816 19785730 PMC2787265

[81] Choe JY, Kim SK. Serum TWEAK as a biomarker for disease activity of systemic lupus erythematosus. Inflamm Res. 2016;65(6):479–88. doi: 10.1007/s00011-016-0930-5 26921306

[82] Dong XW, Zheng ZH, Ding J, Luo X, Li ZQ, Li Y, et al. Combined detection of uMCP-1 and uTWEAK for rapid discrimination of severe lupus nephritis. Lupus. 2018;27(6):971–81. doi: 10.1177/0961203318758507 29451067

[83] Landolt-Marticorena C, Prokopec SD, Morrison S, Noamani B, Bonilla D, Reich H, et al. A discrete cluster of urinary biomarkers discriminates between active systemic lupus erythematosus patients with and without glomerulonephritis. Arthritis Research & Therapy. 2016;18(1):218. doi: 10.1186/s13075-016-1120-0 27716443 PMC5050957

[84] Mirioglu S, Cinar S, Yazici H, Ozluk Y, Kilicaslan I, Gul A, et al. Serum and urine TNF-like weak inducer of apoptosis, monocyte chemoattractant protein-1 and neutrophil gelatinase-associated lipocalin as biomarkers of disease activity in patients with systemic lupus erythematosus. Lupus. 2020;29(4):379–88. doi: 10.1177/0961203320904997 32041504

[85] Selim ZI, Khader TM, Mohammed HO, Al-Hammady DH, Seif HMA, Al-Johi AA. Urinary Tumor Necrosis Factor-Like Weak Inducer of Apoptosis (uTWEAK) as a biomarker for lupus nephritis activity and its correlation with histolopathological findings of renal biopsy. The Egyptian Rheumatologist. 2019;41(1):19–23.

[86] Tan J. The relationship between urinary TWEAK level and the activity of lupus nephritis. http://kns.cnki.net/KCMS/detail/detail.aspx?dbname=CMFD201&file-name=2009240264.nh: Central South University; 2009.

[87] Deshmane SL, Kremlev S, Amini S, Sawaya BE. Monocyte chemoattractant protein-1 (MCP-1): an overview. J Interferon Cytokine Res. 2009;29(6):313–26. doi: 10.1089/jir.2008.0027 19441883 PMC2755091

[88] Du Z, Wu X, Song M, Li P, Wang L. Oxidative damage induces MCP-1 secretion and macrophage aggregation in age-related macular degeneration (AMD). Graefe’s Archive for Clinical and Experimental Ophthalmology. 2016;254(12):2469–76. doi: 10.1007/s00417-016-3508-6 27812755

[89] Kim WK, Choi EK, Sul OJ, Park YK, Kim ES, Yu R, et al. Monocyte chemoattractant protein-1 deficiency attenuates oxidative stress and protects against ovariectomy-induced chronic inflammation in mice. PLoS One. 2013;8(8):e72108. doi: 10.1371/journal.pone.0072108 23977220 PMC3747095

[90] Kumar A, Shalmanova L, Hammad A, Christmas SE. Induction of IL-8(CXCL8) and MCP-1(CCL2) with oxidative stress and its inhibition with N-acetyl cysteine (NAC) in cell culture model using HK-2 cell. Transplant Immunology. 2016;35:40–6. doi: 10.1016/j.trim.2016.02.003 26908203

[91] Abozaid MA, Ahmed GH, Tawfik NM, Sayed SK, Ghandour AM, Madkour RA. Serum and Urine Monocyte Chemoattractant Protein-1 as A Markers for Lupus Nephritis. Egypt J Immunol. 2020;27(1):97–107. 33180392

[92] Gupta R, Yadav A, Aggarwal A. Longitudinal assessment of monocyte chemoattractant protein-1 in lupus nephritis as a biomarker of disease activity. Clin Rheumatol. 2016;35(11):2707–14. doi: 10.1007/s10067-016-3404-9 27624649

[93] Antonelli A, Ferrari SM, Giuggioli D, Ferrannini E, Ferri C, Fallahi P. Chemokine (C-X-C motif) ligand (CXCL)10 in autoimmune diseases. Autoimmun Rev. 2014;13(3):272–80. doi: 10.1016/j.autrev.2013.10.010 24189283

[94] Xu W, Joo H, Clayton S, Dullaers M, Herve MC, Blankenship D, et al. Macrophages induce differentiation of plasma cells through CXCL10/IP-10. J Exp Med. 2012;209(10):1813–23, s1-2. doi: 10.1084/jem.20112142 22987802 PMC3457728

[95] Yates-Binder CC, Rodgers M, Jaynes J, Wells A, Bodnar RJ, Turner T. An IP-10 (CXCL10)-derived peptide inhibits angiogenesis. PLoS One. 2012;7(7):e40812. doi: 10.1371/journal.pone.0040812 22815829 PMC3397949

[96] van Hooij A, Boeters DM, Tjon Kon Fat EM, van den Eeden SJF, Corstjens P, van der Helm-van Mil AHM, et al. Longitudinal IP-10 Serum Levels Are Associated with the Course of Disease Activity and Remission in Patients with Rheumatoid Arthritis. Clin Vaccine Immunol. 2017;24(8). doi: 10.1128/CVI.00060-17 28592626 PMC5583474

[97] Ogawa N, Ping L, Zhenjun L, Takada Y, Sugai S. Involvement of the interferon-gamma-induced T cell-attracting chemokines, interferon-gamma-inducible 10-kd protein (CXCL10) and monokine induced by interferon-gamma (CXCL9), in the salivary gland lesions of patients with Sjögren’s syndrome. Arthritis Rheum. 2002;46(10):2730–41. doi: 10.1002/art.10577 12384933

[98] Scarpini E, Galimberti D, Baron P, Clerici R, Ronzoni M, Conti G, et al. IP-10 and MCP-1 levels in CSF and serum from multiple sclerosis patients with different clinical subtypes of the disease. J Neurol Sci. 2002;195(1):41–6. doi: 10.1016/s0022-510x(01)00680-3 11867072

[99] Hrycek E, Franek A, Błaszczak E, Dworak J, Hrycek A. Serum levels of selected chemokines in systemic lupus erythematosus patients. Rheumatol Int. 2013;33(9):2423–7. doi: 10.1007/s00296-012-2393-5 22461186

[100] Flier J, Boorsma DM, van Beek PJ, Nieboer C, Stoof TJ, Willemze R, et al. Differential expression of CXCR3 targeting chemokines CXCL10, CXCL9, and CXCL11 in different types of skin inflammation. J Pathol. 2001;194(4):398–405. doi: 10.1002/1096-9896(200108)194:4&lt;397::aid-path899&gt;3.0.co;2-s 11523046

[101] Zhang CX, Cai L, Shao K, Wu J, Zhou W, Cao LF, et al. Serum IP-10 is useful for identifying renal and overall disease activity in pediatric systemic lupus erythematosus. Pediatr Nephrol. 2018;33(5):837–45. doi: 10.1007/s00467-017-3867-1 29264699

[102] Marie MA, Abu Khalil RE, Habib HM. Urinary CXCL10: a marker of nephritis in lupus patients. Reumatismo. 2014;65(6):292–7. doi: 10.4081/reumatismo.2013.719 24705033

[103] Abujam B, Cheekatla S, Aggarwal A. Urinary CXCL-10/IP-10 and MCP-1 as markers to assess activity of lupus nephritis. Lupus. 2013;22(6):614–23. doi: 10.1177/0961203313484977 23629827

[104] El-Gohary A, Hegazy A, Abbas M, Kamel N, Nasef SI. Serum and Urinary Interferon-Gamma-Inducible Protein 10 in Lupus Nephritis. J Clin Lab Anal. 2016;30(6):1135–8. doi: 10.1002/jcla.21993 27184880 PMC6806674

[105] Avihingsanon Y, Phumesin P, Benjachat T, Akkasilpa S, Kittikowit V, Praditpornsilpa K, et al. Measurement of urinary chemokine and growth factor messenger RNAs: a noninvasive monitoring in lupus nephritis. Kidney Int. 2006;69(4):747–53. doi: 10.1038/sj.ki.5000132 16518330

[106] Buonafine M, Martinez-Martinez E, Jaisser F. More than a simple biomarker: the role of NGAL in cardiovascular and renal diseases. Clin Sci (Lond). 2018;132(9):909–23. doi: 10.1042/CS20171592 29739822

[107] Nam Y, Kim JH, Seo M, Kim JH, Jin M, Jeon S, et al. Lipocalin-2 protein deficiency ameliorates experimental autoimmune encephalomyelitis: the pathogenic role of lipocalin-2 in the central nervous system and peripheral lymphoid tissues. J Biol Chem. 2014;289(24):16773–89. doi: 10.1074/jbc.M113.542282 24808182 PMC4059121

[108] Moriya H, Mochida Y, Ishioka K, Oka M, Maesato K, Hidaka S, et al. Plasma neutrophil gelatinase-associated lipocalin (NGAL) is an indicator of interstitial damage and a predictor of kidney function worsening of chronic kidney disease in the early stage: a pilot study. Clin Exp Nephrol. 2017;21(6):1053–9. doi: 10.1007/s10157-017-1402-0 28397074

[109] Sun IO, Shin SH, Cho AY, Yoon HJ, Chang MY, Lee KY. Clinical significance of NGAL and KIM-1 for acute kidney injury in patients with scrub typhus. PLoS One. 2017;12(4):e0175890. doi: 10.1371/journal.pone.0175890 28419138 PMC5395225

[110] Walls AB, Bengaard AK, Iversen E, Nguyen CN, Kallemose T, Juul-Larsen HG, et al. Utility of suPAR and NGAL for AKI Risk Stratification and Early Optimization of Renal Risk Medications among Older Patients in the Emergency Department. Pharmaceuticals (Basel). 2021;14(9). doi: 10.3390/ph14090843 34577543 PMC8471084

[111] Qing X, Zavadil J, Crosby MB, Hogarth MP, Hahn BH, Mohan C, et al. Nephritogenic anti-DNA antibodies regulate gene expression in MRL/lpr mouse glomerular mesangial cells. Arthritis Rheum. 2006;54(7):2198–210. doi: 10.1002/art.21934 16804897

[112] Brunner HI, Mueller M, Rutherford C, Passo MH, Witte D, Grom A, et al. Urinary neutrophil gelatinase-associated lipocalin as a biomarker of nephritis in childhood-onset systemic lupus erythematosus. Arthritis Rheum. 2006;54(8):2577–84. doi: 10.1002/art.22008 16868980

[113] Pitashny M, Schwartz N, Qing X, Hojaili B, Aranow C, Mackay M, et al. Urinary lipocalin-2 is associated with renal disease activity in human lupus nephritis. Arthritis Rheum. 2007;56(6):1894–903. doi: 10.1002/art.22594 17530720

[114] Watson L, Tullus K, Pilkington C, Chesters C, Marks SD, Newland P, et al. Urine biomarkers for monitoring juvenile lupus nephritis: a prospective longitudinal study. Pediatr Nephrol. 2014;29(3):397–405. doi: 10.1007/s00467-013-2668-4 24241909

[115] Gómez-Puerta JA, Ortiz-Reyes B, Urrego T, Vanegas-García AL, Muñoz CH, González LA, et al. Urinary neutrophil gelatinase-associated lipocalin and monocyte chemoattractant protein 1 as biomarkers for lupus nephritis in Colombian SLE patients. Lupus. 2018;27(4):637–46. doi: 10.1177/0961203317738226 29073812

[116] Li YJ, Wu HH, Liu SH, Tu KH, Lee CC, Hsu HH, et al. Polyomavirus BK, BKV microRNA, and urinary neutrophil gelatinase-associated lipocalin can be used as potential biomarkers of lupus nephritis. PLoS One. 2019;14(1):e0210633. doi: 10.1371/journal.pone.0210633 30640964 PMC6331123

[117] Sharifipour F, Zeraati A, Sahebari M, Hatef M, Naghibi M, Rezaieyazdi Z, et al. Association of urinary lipocalin-2 with lupus nephritis. Iran J Basic Med Sci. 2013;16(9):1011–5. 24171081 PMC3804839

[118] Susianti H, Iriane VM, Dharmanata S, Handono K, Widijanti A, Gunawan A, et al. Analysis of urinary TGF-β1, MCP-1, NGAL, and IL-17 as biomarkers for lupus nephritis. Pathophysiology. 2015;22(1):65–71. doi: 10.1016/j.pathophys.2014.12.003 25595582

[119] Tawfik Y, Shaat RM, El-Bassiony SR, Hawas S, Effat N. Urinary and serum neutrophil gelatinase-associated lipocalin as a biomarker in Egyptian systemic lupus erythematosus patients: Relation to lupus nephritis and disease activity. The Egyptian Rheumatologist. 2015;37(4, Supplement):S25–S31.

[120] Youssef EM, Ali H, El-Khouly N. Study of urinary neutrophil gelatinase associated lipocalin-2 (uNGAL) as a marker in renal disease activity with systemic lupus erythematosis (lupus nephritis). American Journal of Medicine and Medical Sciences. 2015;5(4):158–63.

[121] Alharazy SM, Kong NC, Mohd M, Shah SA, Abdul Gafor AH, Ba’in A. The role of urinary neutrophil gelatinase-associated lipocalin in lupus nephritis. Clin Chim Acta. 2013;425:163–8. doi: 10.1016/j.cca.2013.07.030 23954775

[122] Elewa EA, El Tokhy MA, Fathy SE, Talaat AM. Predictive role of urinary neutrophil gelatinase-associated lipocalin in lupus nephritis. Lupus. 2015;24(2):138–46. doi: 10.1177/0961203314550225 25199807

[123] Kiani AN, Wu T, Fang H, Zhou XJ, Ahn CW, Magder LS, et al. Urinary vascular cell adhesion molecule, but not neutrophil gelatinase-associated lipocalin, is associated with lupus nephritis. J Rheumatol. 2012;39(6):1231–7. doi: 10.3899/jrheum.111470 22505707 PMC3607283

[124] Maeda-Hori M, Kosugi T, Kojima H, Sato W, Inaba S, Maeda K, et al. Plasma CD147 reflects histological features in patients with lupus nephritis. Lupus. 2014;23(4):342–52. doi: 10.1177/0961203314520840 24474704

[125] Rubinstein T, Pitashny M, Levine B, Schwartz N, Schwartzman J, Weinstein E, et al. Urinary neutrophil gelatinase-associated lipocalin as a novel biomarker for disease activity in lupus nephritis. Rheumatology (Oxford). 2010;49(5):960–71. doi: 10.1093/rheumatology/kep468 20144927 PMC2853702

[126] Satirapoj B, Kitiyakara C, Leelahavanichkul A, Avihingsanon Y, Supasyndh O. Urine neutrophil gelatinase-associated lipocalin to predict renal response after induction therapy in active lupus nephritis. BMC Nephrol. 2017;18(1):263. doi: 10.1186/s12882-017-0678-3 28778196 PMC5545009

[127] Torres-Salido MT, Cortés-Hernández J, Vidal X, Pedrosa A, Vilardell-Tarrés M, Ordi-Ros J. Neutrophil gelatinase-associated lipocalin as a biomarker for lupus nephritis. Nephrol Dial Transplant. 2014;29(9):1740–9. doi: 10.1093/ndt/gfu062 24711435

[128] Yurkovich M, Vostretsova K, Chen W, Avina-Zubieta JA. Overall and cause-specific mortality in patients with systemic lupus erythematosus: a meta-analysis of observational studies. Arthritis Care Res (Hoboken). 2014;66(4):608–16. doi: 10.1002/acr.22173 24106157

[129] Almaani S, Meara A, Rovin BH. Update on Lupus Nephritis. Clin J Am Soc Nephrol. 2017;12(5):825–35. doi: 10.2215/CJN.05780616 27821390 PMC5477208

[130] Whiting PF, Rutjes AW, Westwood ME, Mallett S. A systematic review classifies sources of bias and variation in diagnostic test accuracy studies. J Clin Epidemiol. 2013;66(10):1093–104. doi: 10.1016/j.jclinepi.2013.05.014 23958378

[131] Bullen JA. Studies of Medical Tests: Design and Analytical Considerations. Chest. 2020;158(1s):S103–s12. doi: 10.1016/j.chest.2020.03.006 32658645

[132] Davies JC, Carlsson E, Midgley A, Smith EMD, Bruce IN, Beresford MW, et al. A panel of urinary proteins predicts active lupus nephritis and response to rituximab treatment. Rheumatology (Oxford). 2021;60(8):3747–59. doi: 10.1093/rheumatology/keaa851 33313921 PMC8328509

[133] Fasano S, Pierro L, Borgia A, Coscia MA, Formica R, Bucci L, et al. Biomarker panels may be superior over single molecules in prediction of renal flares in systemic lupus erythematosus: an exploratory study. Rheumatology (Oxford). 2020;59(11):3193–200. doi: 10.1093/rheumatology/keaa074 32211780

[134] Ghasemi M, Kalantari S, Zubarev RA, Nafar M, Saei AA, Heidari SS, et al. Predictive Biomarker Panel in Proliferative Lupus Nephritis- Two-Dimensional Shotgun Proteomics. Iran J Kidney Dis. 2021;1(2):121–33. 33764323

[135] Smith EM, Jorgensen AL, Midgley A, Oni L, Goilav B, Putterman C, et al. International validation of a urinary biomarker panel for identification of active lupus nephritis in children. Pediatr Nephrol. 2017;32(2):283–95. doi: 10.1007/s00467-016-3485-3 27590021 PMC5203828

[136] Su YJ, Lin IC, Wang L, Lu CH, Huang YL, Kuo HC. Next generation sequencing identifies miRNA-based biomarker panel for lupus nephritis. Oncotarget. 2018;9(46):27911–9. doi: 10.18632/oncotarget.25575 29963250 PMC6021342

