# Supplementary material for: Diagnostic test accuracy of novel biomarkers for lupus nephritis—An overview of systematic reviews
Source: PLoS One. 2022 Oct 10;17(10):e0275016. doi: 10.1371/journal.pone.0275016 (PMC9550089; doi:10.1371/journal.pone.0275016)
Supplement: S1 Table — (DOCX) [file pone.0275016.s002.docx]

**S1 Table. Data bases and search strategies used.**

| **Data base** | **Search strategy** |
| --- | --- |
| Pubmed | (("Biomarkers"[Mesh] OR "Biomarker*" OR "Biologic Marker*" OR "Biological Marker*" OR "Laboratory Marker*" OR "Serum Marker*" OR "Surrogate End Point*" OR "Surrogate Endpoint*" OR "Clinical Marker*" OR "Viral Marker*" OR "Biochemical Marker*" OR "Immune Marker*" OR "Immunologic Marker*" OR "Surrogate Marker*" OR "salivary biomarker*" OR "urinary biomarker*" OR "blood biomarker*" OR "Serum biomarker*") OR ("Diagnosis"[Mesh:NoExp] OR "diagnosis" OR "diagnoses" OR "diagnostic" OR "diagnosable" OR "diagnose" OR "diagnosed" OR "diagnosing" OR "screening" OR "monitoring" OR "monitor" OR "test" OR "tests" OR "detection" OR "detecting" OR "signs")) AND ("Lupus Nephritis"[Mesh] OR "Lupus Nephritis" OR "Lupus Glomerulonephritis" OR "Lupus Nephritides") |
| EMBASE | ('biomarker'/exp OR biomarker OR 'biomarkers'/exp OR 'biomarkers' OR 'biologic marker' OR 'biological marker'/exp OR 'biological marker' OR 'laboratory marker' OR 'serum marker' OR 'surrogate end point'/exp OR 'surrogate end point' OR 'surrogate endpoint'/exp OR 'surrogate endpoint' OR 'clinical marker' OR 'viral marker' OR 'biochemical marker'/exp OR 'biochemical marker' OR 'immune marker' OR 'immunologic marker' OR 'surrogate marker'/exp OR 'surrogate marker' OR 'salivary biomarker' OR 'urinary biomarker' OR 'blood biomarker' OR 'serum biomarker' OR 'biologic markers' OR 'biological markers'/exp OR 'biological markers' OR 'laboratory markers' OR 'serum markers' OR 'surrogate end points' OR 'surrogate endpoints' OR 'clinical markers' OR 'viral markers' OR 'biochemical markers' OR 'immune markers' OR 'immunologic markers' OR 'surrogate markers' OR 'exhaled condensate biomarkers' OR 'salivary biomarkers' OR 'urinary biomarkers' OR 'blood biomarkers' OR 'serum biomarkers' OR 'diagnosis'/exp OR diagnosis OR diagnoses OR 'diagnostic'/exp OR diagnostic OR diagnosable OR diagnose OR diagnosed OR diagnosing OR 'screening'/exp OR screening OR 'monitoring'/exp OR monitoring OR 'monitor'/exp OR monitor OR 'test'/exp OR test OR tests OR 'detection'/exp OR detection OR detecting OR signs) AND ('lupus nephritis'/exp OR 'lupus nephritis' OR 'lupus glomerulonephritis'/exp OR 'lupus glomerulonephritis' OR 'lupus nephritides') |
| WOS | TS=(Biomarker* OR "Biologic Marker*" OR "Biological Marker*" OR "Laboratory Marker*" OR "Serum Marker*" OR "Surrogate End Point*" OR "Surrogate Endpoint*" OR "Clinical Marker*" OR "Viral Marker*" OR "Biochemical Marker*" OR "Immune Marker*" OR "Immunologic Marker*" OR "Surrogate Marker*" OR "salivary biomarker*" OR "urinary biomarker*" OR "blood biomarker*" OR "Serum biomarker*" OR diagnosis OR diagnoses OR diagnostic OR diagnosable OR diagnose OR diagnosed OR diagnosing OR screening OR monitoring OR monitor OR test OR tests OR detection OR detecting OR signs) AND TS=("Lupus Nephritis" OR "Lupus Glomerulonephritis" OR "Lupus Nephritides") |
| Scopus | TITLE-ABS-KEY("Lupus Nephritis" OR "Lupus Glomerulonephritis" OR "Lupus Nephritides") AND TITLE-ABS-KEY(Biomarker* OR "Biologic Marker*" OR "Biological Marker*" OR "Laboratory Marker*" OR "Serum Marker*" OR "Surrogate End Point*" OR "Surrogate Endpoint*" OR "Clinical Marker*" OR "Viral Marker*" OR "Biochemical Marker*" OR "Immune Marker*" OR "Immunologic Marker*" OR "Surrogate Marker*" OR "salivary biomarker*" OR "urinary biomarker*" OR "blood biomarker*" OR "Serum biomarker*" OR diagnosis OR diagnoses OR diagnostic OR diagnosable OR diagnose OR diagnosed OR diagnosing OR screening OR monitoring OR monitor OR test OR tests OR detection OR detecting OR signs) |
| Lilacs | tw:((tw:(biomarker* OR "Biologic Marker*" OR "Biological Marker*" OR "Laboratory Marker*" OR "Serum Marker*" OR "Surrogate End Point*" OR "Surrogate Endpoint*" OR "Clinical Marker*" OR "Viral Marker*" OR "Biochemical Marker*" OR "Immune Marker*" OR "Immunologic Marker*" OR "Surrogate Marker*" OR "salivary biomarker*" OR "urinary biomarker*" OR "blood biomarker*" OR "Serum biomarker*" OR diagnosis OR diagnoses OR diagnostic OR diagnosable OR diagnose OR diagnosed OR diagnosing OR screening OR monitoring OR monitor OR test OR tests OR detection OR detecting OR signs OR diagnóstico OR diagnose OR rastreamento OR sinais OR triagem OR teste OR detecção OR monitoramento OR diagnosis OR rastreo OR señales OR tamizaje OR prueba OR detección OR monitorización OR monitoreo OR biomarcadores OR "Marcadores Biológicos" OR "Marcadores Bioquímicos" OR "Marcadores Clínicos" OR "Marcadores Imunológicos" OR "Marcadores Substitutos" OR "Marcadores Séricos" OR "Marcadores Virais" OR "Marcadores de Laboratorio" OR "Marcadores de Soro" OR "Marcadores Inmunológicos" OR "Marcadores Sustitutos" OR "Marcadores Virales")) AND (tw:("Lupus Nephritis" OR "Lupus Glomerulonephritis" OR "Lupus Nephritides" OR "Nefrite Lúpica" OR "Glomerulonefrite Lúpica" OR "Nefritis Lúpica"))) AND ( db:("LILACS")) |
| Cochrane | (Biomarker* OR "Biologic Marker*" OR "Biological Marker*" OR "Laboratory Marker*" OR "Serum Marker*" OR "Surrogate End Point*" OR "Surrogate Endpoint*" OR "Clinical Marker*" OR "Viral Marker*" OR "Biochemical Marker*" OR "Immune Marker*" OR "Immunologic Marker*" OR "Surrogate Marker*" OR "salivary biomarker*" OR "urinary biomarker*" OR "blood biomarker*" OR "Serum biomarker*" OR diagnosis OR diagnoses OR diagnostic OR diagnosable OR diagnose OR diagnosed OR diagnosing OR screening OR monitoring OR monitor OR test OR tests OR detection OR detecting OR signs):ti,ab,kw AND ("Lupus Nephritis" OR "Lupus Glomerulonephritis" OR "Lupus Nephritides"):ti,ab,kw |
| OpenGrey | ("Lupus Nephritis" OR "Lupus Glomerulonephritis" OR "Lupus Nephritides") AND (Biomarker* OR "Biologic Marker*" OR "Biological Marker*" OR "Laboratory Marker*" OR "Serum Marker*" OR "Surrogate End Point*" OR "Surrogate Endpoint*" OR "Clinical Marker*" OR "Viral Marker*" OR "Biochemical Marker*" OR "Immune Marker*" OR "Immunologic Marker*" OR "Surrogate Marker*" OR "salivary biomarker*" OR "urinary biomarker*" OR "blood biomarker*" OR "Serum biomarker*" OR diagnosis OR diagnoses OR diagnostic OR diagnosable OR diagnose OR diagnosed OR diagnosing OR screening OR monitoring OR monitor OR test OR tests OR detection OR detecting OR signs) |
| ProQuest (thesis and dissertations) | noft(Biomarker* OR "Biologic Marker*" OR "Biological Marker*" OR "Laboratory Marker*" OR "Serum Marker*" OR "Surrogate End Point*" OR "Surrogate Endpoint*" OR "Clinical Marker*" OR "Viral Marker*" OR "Biochemical Marker*" OR "Immune Marker*" OR "Immunologic Marker*" OR "Surrogate Marker*" OR "salivary biomarker*" OR "urinary biomarker*" OR "blood biomarker*" OR "Serum biomarker*" OR diagnosis OR diagnoses OR diagnostic OR diagnosable OR diagnose OR diagnosed OR diagnosing OR screening OR monitoring OR monitor OR test OR tests OR detection OR detecting OR signs) AND noft("Lupus Nephritis" OR "Lupus Glomerulonephritis" OR "Lupus Nephritides") |

WOS = Web of Science.
