# Supplementary material for: Diagnostic test accuracy of novel biomarkers for lupus nephritis—An overview of systematic reviews
Source: PLoS One. 2022 Oct 10;17(10):e0275016. doi: 10.1371/journal.pone.0275016 (PMC9550089; doi:10.1371/journal.pone.0275016)
Supplement: S2 Table — (PDF) [file pone.0275016.s003.pdf]

**S2 Table. Overview of reported cut-off thresholds used on primary studies in the included SRs.**

| Author (year) | Biomarker | Method used | Cut-off positive result | Number of primary studies |
|---|---|---|---|---|
| Eggleton P. et al. (2014) | anti-C1q | ELISA | > 8 U/ml | 1 |
| | | | > 10 U/ml | 1 |
| | | | >15 U/ml | 1 |
| | | | > 20 U/ml | 6 |
| | | | > 20 U/ml + 5 SD above controls | 2 |
| | | | > 32 U/ ml | 3 |
| | | | > 40 U/ml | 3 |
| | | | > 80 U/ml | 2 |
| | | | > 90U/ml | 2 |
| | | | > 137 U/ml | 1 |
| | | | > 50 mcg/ml anti-C1q | 1 |
| | | | > mean OD + 2 SD above 30 inactive SLE controls | 1 |
| | | | > mean OD + 5 SD above 30 controls | 1 |
| | | | > mean 95% OD above 59 HC controls | 1 |
| | | | > mean OD + 2SD above 63 controls | 1 |
| | | | > mean OD + 2SD above 100 controls | 1 |
| Fang Y. G. et al. (2020) | uNGAL | ELISA | 0.3 ng/mg Cr | 1 |
| | | | 0.4 ng/mgCr | 1 |
| | | | 0,6 ng/mg Cr | 2 |
| | | | 0.8 ng/mg Cr | 1 |
| | | | 10 ng/mg Cr | 1 |
| | | | 91 ng/ mg Cr | 1 |
| | | | 11 ng/ml | 2 |
| | | | 13 ng/ml | 2 |
| | | | 28 ng/ml | 1 |
| | | | 30 ng/ml | 1 |
| | | | 0.3 ng/mg Cr | 1 |

| | | | | |
|---|---|---|---|---|
| Gao, Y. et al. (2020) | uNGAL | ELISA | 0.4 ng/mgCr | 1 |
| | | | 0,6 ng/mg Cr | 2 |
| | | | 10 ng/mg Cr | 1 |
| | | | 20 ng/mg Cr | 2 |
| | | | 39 ng/mg Cr | 1 |
| | | | 91 ng/ mg Cr | 1 |
| | | | 11 ng/ml | 3 |
| | | | 13 ng/ml | 3 |
| | | | 13 ng/dl | 1 |
| | | | 18 ng/ml | 1 |
| | | | 28 ng/ml | 1 |
| | | | 30 ng/ml | 1 |
| | | | 80 ng/ml | 1 |
| | | | 446 pg/ml | 1 |
| Ma, H. Y. et al (2021) | TWEAK | ELISA (serum) | 395 pg/ml | 1 |
| | | | 1542 ng/ml | 1 |
| | | ELISA (urine) | 4.3 pg/ mg Cr | 1 |
| | | | 4.9 pg/ mg Cr | 1 |
| | | | 8.2 pg/ mg Cr | 1 |
| | | | 9.1 pg/ mg Cr | 1 |
| | | | 12.5 pg/ mg Cr | 1 |
| | | | 13 pg/ mg Cr | 1 |
| | | | 26.9 pg/ mg Cr | 1 |

OD= optical density; Cr= urinary creatinine.
